# N1-Methyladenosine (m1A) Regulation Associated With the Pathogenesis of Abdominal Aortic Aneurysm Through *YTHDF3* Modulating Macrophage Polarization

**DOI:** 10.3389/fcvm.2022.883155

**Published:** 2022-05-10

**Authors:** Yihao Wu, Deying Jiang, Hao Zhang, Fanxing Yin, Panpan Guo, Xiaoxu Zhang, Ce Bian, Chen Chen, Shuixin Li, Yuhan Yin, Dittmar Böckler, Jian Zhang, Yanshuo Han

**Affiliations:** ^1^School of Life and Pharmaceutical Sciences, Dalian University of Technology, Panjin, China; ^2^Department of Vascular Surgery, Dalian Municipal Central Hospital, Dalian, China; ^3^Department of Cardiovascular Surgery, The General Hospital of the PLA Rocket Force, Beijing, China; ^4^School of Biomedical Sciences, University of Queensland, St Lucia, Brisbane, QLD, Australia; ^5^Department of Vascular and Endovascular Surgery, University Hospital Heidelberg, Heidelberg, Germany; ^6^Department of Vascular Surgery, The First Hospital of China Medical University, Shenyang, China

**Keywords:** abdominal aortic aneurysm (AAA), N1-Methyladenosine (m1A) regulation, immune infiltration, *YTHDF3*, macrophages, M1/M2 polarization, RNA immunoprecipitation-sequencing (RIP-Seq)

## Abstract

**Objectives:**

This study aimed to identify key AAA-related m1A RNA methylation regulators and their association with immune infiltration in AAA. Furthermore, we aimed to explore the mechanism that m1A regulators modulate the functions of certain immune cells as well as the downstream target genes, participating in the progression of AAA.

**Methods:**

Based on the gene expression profiles of the GSE47472 and GSE98278 datasets, differential expression analysis focusing on m1A regulators was performed on the combined dataset to identify differentially expressed m1A regulatory genes (DEMRGs). Additionally, CIBERSORT tool was utilized in the analysis of the immune infiltration landscape and its correlation with DEMRGs. Moreover, we validated the expression levels of DEMRGs in human AAA tissues by real-time quantitative PCR (RT-qPCR). Immunofluorescence (IF) staining was also applied in the validation of cellular localization of *YTHDF3* in AAA tissues. Furthermore, we established LPS/IFN-γ induced M1 macrophages and *ythdf3* knockdown macrophages *in vitro*, to explore the relationship between *YTHDF3* and macrophage polarization. At last, RNA immunoprecipitation-sequencing (RIP-Seq) combined with PPI network analysis was used to predict the target genes of *YTHDF3* in AAA progression.

**Results:**

Eight DEMRGs were identified in our study, including *YTHDC1, YTHDF1-3, RRP8, TRMT61A* as up-regulated genes and *FTO, ALKBH1* as down-regulated genes. The immune infiltration analysis showed these DEMRGs were positively correlated with activated mast cells, plasma cells and M1 macrophages in AAA. RT-qPCR analysis also verified the up-regulated expression levels of *YTHDC1, YTHDF1*, and *YTHDF3* in human AAA tissues. Besides, IF staining result in AAA adventitia indicated the localization of *YTHDF3* in macrophages. Moreover, our *in-vitro* experiments found that the knockdown of *ythdf3* in M0 macrophages inhibits macrophage M1 polarization but promotes macrophage M2 polarization. Eventually, 30 key AAA-related target genes of *YTHDF3* were predicted, including *CD44, mTOR, ITGB1, STAT3*, etc.

**Conclusion:**

Our study reveals that m1A regulation is significantly associated with the pathogenesis of human AAA. The m1A “reader,” *YTHDF3*, may participate in the modulating of macrophage polarization that promotes aortic inflammation, and influence AAA progression by regulating the expression of its target genes.

## Introduction

Abdominal Aortic Aneurysm (AAA) is a common vascular disease featured with the pathological dilatation of the abdominal aorta and the consistent weakening of the aortic wall (1). Currently in clinical cases, male patients with AAA diameter >5.5 cm, female patients with AAA diameter >5.0 cm as well as ruptured AAAs are only recommended to endovascular aneurysm repair (EVAR) or open surgical repair (OSR) (2, 3), due to the extreme lack of effective drug therapy (4). The Multi-center Aneurysm Screening Study (MASS) in 2012, a randomized controlled trial (RCT) of screening for AAA in a large UK population, showed a prevalence rate of an aortic aneurysm of 4.9% (5). Recent study has reported an AAA prevalence rate at 1.3–5% of screened men 65 years and older (6). However, once an AAA is detected, the average life expectancy for males is 11 years (7). Meanwhile, a ruptured AAA (rAAA) will result in a mortality rate of up to 81% for men (6).

Since the risks and limitations of surgical repairs in AAA treatment, together with the high mortality of rAAA are non-negligible and concerning, many fundamental researches have focused on the etiology of AAA in order to specifically inhibit the progression of AAA. Current theories on AAA pathogenesis indicate that AAA is mainly characterized by acquired immune-driven destruction of the aortic wall and a response to atherosclerosis and thrombosis, which cause the subsequent extracellular matrix (ECM) degradation, vascular inflammation, apoptosis of vascular smooth muscle cells (VSMCs) and abnormal oxidative stress response (8, 9). Moreover, inherited factors in AAA pathogenesis are gaining increasing attention from researchers nowadays. For example, significant single nucleotide polymorphisms (SNPs) related to AAA are identified in genome-wide association studies, proposing affected genes or proteins like IL-6 receptor, LDL receptor, *SMYD3* and *MMP-9* (8). *mTOR*Scholars also found that *miR-424/322* provide protection against AAA formation by modulating the Smad2/3/runt-related transcription factor 2 axis, which implies the potential roles of epigenetic regulation in AAA progression (10).

Epigenetic regulation, including DNA methylation, RNA methylation, non-coding RNAs (ncRNAs), histone acetylation and deacetylation, etc., refers to regulating gene expression and structure without altering genome sequence, and thus affect human health and disease (11). Additionally, recent studies have shown that epigenetics is strongly associated with various vascular diseases. At chromatin level, study has demonstrated that histone acetylation and histone acetyltransferases play significant roles in human AAA (12). At mRNA level, increased N6-methyladenosine (m6A) modification level, 5-methylcytosine (m5C) methylation level and the up-regulating expression of their regulatory genes were verified in human AAA (13, 14). At ncRNAs level, Li et al. illustrated that *miR-124a* can involve in the regulation of Wnt/β-Catenin and P53 pathways to inhibit AAA *via* targeting *BRD4* (15). Meanwhile, circular RNA expression and its potential regulation in AAA have attracted widespread attention as well (16).

Based on the existing findings of epigenetic regulation in AAA, we started to focus on other types of RNA methylation modifications in AAA, especially N1-Methyladenosine (m1A) modification. M1A modification changes the secondary structure of RNAs by adding a methyl group to the N1 position of adenosine, thus affecting the interaction between RNAs and proteins (17). M1A methylation is found in rRNAs, tRNAs, mRNAs and mitochondrial RNAs, regulated by three types of m1A regulators: First, m1A “writers,” including *TRMT6*/*TRMT61A, TRMT61B, TRMT10C, BMT2* and *RRP8*, work as methyltransferases to add m1A modification on RNAs; Second, m1A “erasers,” containing *FTO, ALKBH1-3*, can eliminate the modification of m1A; Third, m1A “readers,” comprising *YTHDF1-3* and *YTHDC1*, serve as RNA binding proteins which can read m1A methylation modification information as well as recognizing and binding to m1A methylation sites (17–19). Although m1A modification patterns have been reported in a variety of diseases, involving Ovarian Cancer, Pancreatic Cancer and Oral Squamous Cell Carcinoma (19–21), there are currently no relevant reports of m1A modification and regulation in AAA, motivating us to perform research on the role of m1A regulation in AAA.

In addition, inflammatory immune cell infiltration in aortic adventitia is a key characteristic of AAA (22). To be specific, studies have shown that macrophages play an essential role in the formation and development of AAA (23). Furthermore, the transition (also known as macrophage polarization) from M0 macrophages to pro-inflammatory M1 phenotype macrophages or anti-inflammatory M2 phenotype macrophages has been proved to have a regulatory effect on the vascular inflammation of AAA (24). It is worth mentioning that various epigenetic mechanisms are associated with macrophage polarization and can further regulate the inflammation in cardiometabolic and vascular diseases (25). Song et al. also confirmed that circular RNA Cdyl promotes AAA formation by inducing M1 macrophage polarization and M1-type inflammation (26), inspiring the idea of exploring and utilizing specific epigenetic mechanism to regulate macrophage polarization in AAA.

This study aimed to explore the possible epigenetic mechanism of m1A regulation in the pathogenesis of AAA, contributing to the development of AAA etiology from the perspective of m1A epigenetic regulation. First of all, our study used bioinformatics analysis and experimental validation at human tissue level to establish the research basis of the expression pattern of m1A regulators and their correlation with immune cells in AAA. Secondly, based on this research foundation, we chose to focus on *YTHDF3*, the m1A “reader,” and further explored its regulation of macrophage polarization in *ex-vivo* experiments. At last, RIP-Seq analysis of *YTHDF3* combined with bioinformatics analysis was conducted to predict the AAA-related target genes of *YTHDF3*.

## Materials and Methods

### Data Sources and Screening of AAA-Related m1A Regulators

The gene expression profiling datasets GSE47472 and GSE98278 were acquired from the GEO database (https://www.ncbi.nlm.nih.gov/geo/) for analysis. Among them, GSE47472 included mRNA expression data from 14 AAA neck specimens and 8 normal aortic specimens of organ donors, while GSE98278 contained 48 AAA samples but no normal aortic samples. All data from these two chips were free to use and both of the chips were annotated with the same platform named Illumina HumanHT-12 V4.0 Expression Beadchip (https://www.ncbi.nlm.nih.gov/geo/query/acc.cgi?acc=GPL10558). Thereafter, the raw data from these two datasets underwent data combination, background correction and quantile normalization by “limma” package in R. After preprocessing, the combined dataset consisted of 8 normal aortic samples and 62 AAA samples altogether, which was prepared for further analysis.

The overall differential gene expression analysis based on the combined dataset was performed with the statistical threshold of |*log*_2_*Fold Change (FC)*|≥1 and adjusted *P value* <0.05 also by the “limma” R package. In this study, fourteen genes extracted from formal relevant reviews and research articles, including *YTHDF1, YTHDF2, YTHDF3, RRP8, ALKBH1, ALKBH2, ALKBH3, YTHDC1, TRMT6, TRMT61A, TRMT61B, TRMT10C, FTO* and *BMT2*, were regarded as common m1A RNA methylation regulators. Then, the differentially expressed m1A regulatory genes (DEMRGs) were identified *via* the overlapping of differentially expressed genes (DEGs) and 14 candidate m1A regulators. Due to their significantly differential expression level in AAAs, DEMRGs were also defined as AAA-Related m1A regulators.

### Co-expression Analysis and Functional Annotation of AAA-Related m1A Regulators

Based on the DEMRGs and our combined dataset, co-expression analysis aiming to dig the co-expressed genes of DEMRGs in AAA tissues was carried out with “limma” and “Hmisc” packages within R. To be specific, the co-expression analysis was conducted using Pearson correlation as well as the cut-off criteria of |R| ≥ 0.8 and *P* < 0.05. After that, among the union set of co-expressed genes of all DEMRGs, we performed Gene Ontology (GO) enrichment analysis, which included terms of biological processes (BP), cellular components (CC), molecular functions (MF), and Kyoto Encyclopedia of Genes and Genomes (KEGG) pathway enrichment analysis utilizing “clusterProfiler” R package. The adjusted *P* < 0.05 together with *q value* < 0.05 was considered statistically significant and 15 terms with the top gene counts of each enrichment category were displayed.

A LASSO model was established to primarily screen the qualified biomarkers from AAA-Related m1A regulators in the diagnosis of AAA. The “glmnet” package in R software was used to establish the LASSO regression model (family = “binomial”) based on the gene expression profiles of DEMRGs and the status (AAA or normal) of all the samples. In the current study, the minimum lambda value (λ.min) judged by the minimum binomial de*via*nce was used as a reference to identify the essential genes to be included in the AAA diagnostic model. Additionally, key genes obtained from the LASSO model were then used to conduct Receiver Operating Characteristic (ROC) curve analysis in order to quantitatively evaluate the diagnostic value of DEMRGs in AAA. The ROC curve model was established with HIPLOT online tools (https://hiplot.com.cn/basic/roc) and genes with Area Under Curve (AUC) more than 90% were reckoned as ideal diagnostic biomarkers in identifying AAA.

### Analysis of Immune Cell Infiltration in AAA and the Correlation Between AAA-Related m1A Regulators and Immune Cells

Because inflammatory immune cell infiltration was a substantial characteristic of AAA pathology, we performed analysis of immune cell infiltration in AAA with CIBERSORT tool in R. CIBERSORT algorithm is an analytical tool from the Alizadeh Lab developed by Newman et al. to provide an estimation of the abundances of member cell types in a mixed cell population, using gene expression data (27). All the relevant R packages and files could be acquired from the CIBERSORT web portal (http://cibersort.stanford.edu/). The gene expression matrix of our combined dataset was input to R and the leukocyte signature matrix (LM22), which contains 547 genes that distinguish 22 human hematopoietic cell phenotypes, was selected as the signature gene file. Afterwards, the result of immune cell infiltration landscape in AAA and normal aortic tissue was output. Then, immune cell types with zero abundance value in more than half of the samples were regarded as extremely low proportion and were thus excluded from our research. The adjusted *P* < 0.05 was set as the threshold for statistical significance. The relative contents of diverse immune cell types in each sample were visualized as heat maps according to the research outcomes. Moreover, specific immune cell types with significant differences between AAA and normal samples were individually visualized as box plots.

Pearson correlation analysis was also performed to further explore the role of AAA-related m1A regulators during the development of AAA, participating in immune cell infiltration. The degree of correlation among immune cells was analyzed according to the relative proportion of immune cells in each sample. Besides, the relative content of each immune cell type and the expression data of DEMRGs were used to explore the relationship between AAA-Related m1A regulators and immune cells. The Pearson correlation coefficient (R) ≥ 0.45 together with adjusted *P* < 0.05 was considered as a significantly strong correlation.

### Clinical Specimens Collection and Preprocessing

Our study obtained human AAA tissue from the clinical cases at the Department of Vascular Surgery, The First Hospital of China Medical University (CMU), undergoing the elective open surgical repair for aneurysm as previously described (14, 28). The AAA population selection and biopsy tissue collection were conducted by China Medical University Aneurysm Biobank (CMU-aB). However, patients with Marfan syndrome, Ehlers-Danlos syndrome, and other identified vascular or connective tissue disorders were excluded from the study. For the inclusive 141 AAA patients in CMU-aB, AAA was diagnosed by computed tomography angiography (CTA). All human AAA samples collection was conducted according to the guidelines from the World Medical Association Declaration of Helsinki. Meanwhile, normal abdominal aorta samples from 18 organ donors were used as the control tissue samples and this was conducted in accordance with the Declaration of Istanbul. Exclusion criteria for the control group included drug history, cancer, infection, and any additional immune-related diseases that might have negatively influenced the study. In addition, each case provided the informed consent for participation. Afterwards, we selected a total of 63 fresh AAA samples and 18 control samples to form our aneurysm biobank applied in this study. The Ethics Committees of the First Hospital of China Medical University approved our study protocol (ethical approval number: 2019-97-2).

All tissue samples were divided into two parts according to different experimental purposes and stored at −80°C condition. The first part consisted of 35 AAA samples and 8 control samples, which was used for total RNA extraction and subsequent reverse transcription PCR as well as RT-qPCR analysis. The second part contained 28 AAA samples and 10 control samples, prepared for protein extraction and quantitative Western blot (WB) analysis.

For the first part of tissue samples, total mRNA was extracted from aortic tissues utilizing TRIzol reagent (93289, Sigma-Aldrich, USA) according to the standard protocol. To ensure the accuracy of the subsequent experiments, only high-quality RNAs with their A260/A280 ratio >1.8 and <2.2 were qualified for use. Thereafter, PrimeScript™ RT Master Mix (RR036A, TaKaRa Bio, Shiga, Japan) was used to synthesize cDNA from total mRNA with the procedure of 15 min of reverse transcription reaction under 37°C followed by 5 s of inactivation of reverse transcriptase under 85°C. For the other part of tissue samples, proteins were extracted from fresh frozen tissues through RIPA lysis buffer (P0013B, Beyotime, Shanghai, China) and later the concentration of each sample was quantified by BCA Protein Assay kit (P0012, Beyotime, Shanghai, China). At last, all the measured protein samples were mixed with 5 ×SDS-PAGE Sample Loading Buffer (P0015, Beyotime, Shanghai, China) in a 4:1 volume ratio. All the cDNA and protein samples undergone preprocessing were then stored at −20°C.

Moreover, the typical 2–3 μm AAA tissue sections utilized to carry out Immunofluorescence (IF) staining analysis were acquired from FangDa Mass Hospital in Yingkou City, Liaoning Province.

### Quantitative Real-Time Polymerase Chain Reaction (RT-qPCR) Analysis

RT-qPCR method was applied in the detection of gene relative expression on mRNA level. For all of the tissue-derived samples and cell-derived samples included in the RT-qPCR analysis, the TB Green^®^ Premix Ex Taq ™ II plus Tli RNaseH (RR820A, TaKaRa Bio, Shiga, Japan) was utilized to conduct RT-qPCR in Applied Biosystems 7500 Real-Time PCR System (Thermo Fisher Scientific, USA) in accordance with the modified two-step amplification protocol. The RT-qPCR conditions were as follows: 30 s of initial denaturation under 95°C; followed by 5 s of denaturation under 95°C, and 34 s of annealing combined with extension under 60°C for 50 cycles. Housekeeping gene glyceraldehyde 3-phosphate dehydrogenase (*GAPDH*) served as the internal control in every RT-qPCR test for normalization. Each pair of forward and reverse primers was provided by Sangon Biotech (Shanghai, China) and their sequences are shown in Table 1. The relative expression levels of mRNAs in samples were calculated with the 2^−ΔΔCT^ method.

**Table 1:** Primer sequences used for RT-qPCR.

**Gene**	**Category**	**Primer sequences (5^**′**^to 3^**′**^)**
*TRMT61A*	m1A “writer”	F: GTACCCCTACCCCTCACAGA R: CCTGAATGCACCTCCCCTAC
*RRP8*	m1A “writer”	F: CAGTGGTAAGAGGTTGCTCCAT R: TGGTATGCTCTTCCCTCTGC
*YTHDC1*	m1A “reader”	F: TCTTCCGTTCGTGCTGTCC R: GGACCATACACCCTTCGCTT
*YTHDF1*	m1A “reader”	F:TGGACACCCAGAGAACAAAAGG R:TGAGGTATGGAATCGGAGGGT
*YTHDF2*	m1A “reader”	F: AGTGTCAGGGACAAAAGCCTCC R: TTTTGGTCTCTGCTCCAAGAGG
*YTHDF3*-Homo sapiens	m1A “reader”	F: TAGGGAGTCTGTCCGCCATT R: GACATTCTTCACCGCAACCC
*GAPDH*-Homo sapiens	internal reference	F: GTTGGAGGTCGGAGTCAACGG R: GAGGGATCTCGCTCCTGGAGGA
*YTHDF3*-Mus musculus	m1A “reader”	F: CAGAGACCTAAAGGGCAAGGA R: CATGCTGCTTCCCCAAGAGA
*GAPDH*-Mus musculus	internal reference	F: CAGCTACTCGCGGCTTTAC R: TTCACACCGACCTTCACCATT
*CD86*-Mus musculus	M1 macrophage marker	F: CAGCACGGACTTGAACAACC R: TGTGCCCAAATAGTGCTCGT
*iNOS*-Mus musculus	M1 macrophage marker	F: TGCCAGGGTCACAACTTTACA R: CAGCTCAGTCCCTTCACCAA
*TNFα*-Mus musculus	M1 macrophage marker	F: GATCGGTCCCCAAAGGGATG R: GTTTGCTACGACGTGGGCT
*CD206*-Mus musculus	M2 macrophage marker	F: GCACTGGGTTGCATTGGTTT R: CCTGAGTGGCTTACGTGGTT
*Arg1*-Mus musculus	M2 macrophage marker	F: GTGAAGAACCCACGGTCTGT R: AGAAAGGACACAGGTTGCCC
*TGFβ*-Mus musculus	M2 macrophage marker	F: GATACGCCTGAGTGGCTGTC R: TTTGGGGCTGATCCCGTTG

*F, forward; R, reverse; iNOS, inducible nitric oxide synthase; TNF, tumor necrosis factor; Arg, arginine; TGF, transforming growth factor*.

### Western Blot (WB) Analysis

Western Blot (WB) staining has been widely used in the analysis of gene relative expression on protein level. Protein samples (20 μg of each lane) were separated by sodium dodecyl sulfate-polyacrylamide gel electrophoresis (SDS-PAGE) using 5% spacer gel and 12% separation gel, then transferred onto polyvinylidene difluoride (PVDF) membrane. The membranes were blocked (5% skimmed milk powder in 1 ×TBS with 0.05% Tween 20) for 2 h, followed by incubation with primary antibody at a dilution of 1:1,000 for rabbit anti-*YTHDF3* (K005805P, Solarbio, Beijing, China), anti-*iNOS* (BA0362, BOSTER, Wuhan, China) and 1:10,000 for rabbit anti-*GAPDH* (D110016, Sangon Biotech, Shanghai, China), anti-β*-Actin* (abs132001, absin, Shanghai, China), overnight at 4°C. The blots were then incubated with Horseradish Peroxidase (HRP)-labeled Goat Anti-Rabbit IgG (A0208, Beyotime, Shanghai, China) at a dilution of 1:1,000 for 1–2 h at room temperature (RT). After washing the membranes 3 times with Tris-buffered saline with Tween (TBST), immunoreactive bands were developed and detected using hypersensitive Electro Chemical Luminescence (ECL) kit (P1000-100, Applygen, Beijing, China) and the chemiluminescence detection system (FluorChem HD2, ProteinSimple, USA). The densitometry was performed with Image J software 1.53 (W. Rasband, Research Services Branch, NIMH, National Institutes of Health, Bethesda, MD, USA) and normalized to the signal intensity of *GAPDH* or β*-Actin* for equal protein loading control of each sample in each experiment.

### Immunofluorescence (IF) Double Staining Analysis of *CD68* and *YTHDF3* in Human AAA Tissue

Since our bioinformatics analysis of immune cell infiltration in AAA revealed a strong correlation between macrophages and *YTHDF3*, IF double staining analysis of *CD68* (surface marker of macrophages) and *YTHDF3* was carried out for cellular localization in AAA tissue.

Firstly, typical 2–3 μm paraffin-embedded AAA sections were dewaxed and rehydrated in line with xylene, isopropanol and descending gradient ethanol. Then, the process of antigen epitopes retrieving was performed by boiling the sections within 0.01M citrate buffer (pH = 6.0). After rinsing by Tris-buffered saline (TBS), the exposed tissues were blocked by Immuno Staining Blocking Buffer (P0102, Beyotime, Shanghai, China) for 2 h, followed by incubation overnight using the rabbit anti-*YTHDF3* antibody (dilution 1:100; K005805P, Solarbio, Beijing, China) at 4°C in the humidified chamber. After washing by TBS thrice, the Cy3 (red) conjugated Goat Anti-Rabbit IgG (dilution 1:400; GB21303, Servicebio, Wuhan, China) was utilized to incubate the sections for 2 h under RT, light-free condition. Afterwards, sections were washed 3 times with fresh TBS, and incubated with rabbit anti-*CD68* antibody (dilution 1:200; abs120102, absin, Shanghai, China) overnight under 4°C in the humid chamber. Being rinsed thrice with TBS, sections were incubated with Goat anti-Rabbit IgG-AlexaFluor 488 (green, dilution 1:400; abs20025, absin, Shanghai, China) at RT condition away from light for another 2 h. The sections were finally washed three times with TBS, and stained by Antifade Mounting Medium with DAPI (P0131, Beyotime, Shanghai, China) for 10 min in a dark chamber. At last, the fluorescence microscope (Nikon, Tokyo, Japan) was used to observe sections and acquire photos of different visual fields.

### Cell Culture and Induction of M1 Polarization

The murine macrophage cell line RAW264.7 was obtained from Fudancell (FuHeng BioLogy, FH0328, Shanghai, China) and cultured in 10 cm plates with complete growth medium DMEM High Glucose (MA0545, meilunbio, Dalian, China) containing 10% fetal bovine serum (10100-147, Gibco, Thermo Fisher Scientific, USA) and 1% Penicillin/Streptomycin (10,000 U/mL; SV30010, HyClone, USA) in humidified air with 5% CO_2_ at 37°C. To inhibit the differentiation of RAW264.7 cell line, cell scrapers were used for cell subculturing instead of 0.25% Trypsin-EDTA.

Untreated RAWs were defined as M0 macrophages. 1 × 10^6^ M0 macrophages were seeded into each chamber of the 6-well plates for 12–24 h. When the cells were adherent, the previous complete growth medium was removed and treatment for M0 macrophages was placed as follows: control group, 2 mL of complete growth medium for each well only; M1 polarization group was induced by 100 ng/mL lipopolysaccharide (LPS; SMB00610, Sigma-Aldrich, USA) and 50 ng/mL recombinant murine interferon-gamma (IFN-γ; C600059, Sangon Biotech, Shanghai, China) with a total medium mixture volume of 2 mL. After applying the above intervention conditions, the cells were further cultured at 37°C and 5% CO_2_ for 24 h, followed by total RNA extraction, protein extraction and cell culture supernatant collection according to the protocols mentioned in parts 2.4 and 2.10.

### *YTHDF3* siRNA Transfection and Further Induction of M1 Polarization

Small interfering RNA (siRNA) against mouse *YTHDF3* as well as Negative Control (NC) siRNA was designed and synthesized by RiboBio (Guangzhou, China). The three siRNA sequences arranged for *YTHDF3* mRNA silencing were *ythdf3*-si-1(5′-3′): ACUCAUUGGUUCCUUUAAGGG; (3′-5′): CUUAAAGGAACCAAUGAGUCC; *ythdf3*-si-2 (5′-3′): UGGAUUUGUACCAUUCUUCUG; (3′-5′): GAAGAAUGGUACAAAUCCAAG; *ythdf3*-si-3 (5′-3′): UCAUAUUCUGAAUCUCAUCCU; (3′-5′): GAUGAGAUUCAGAAUAUGAAG.

RAW264.7 cells were transfected with 50 nM *YTHDF3* siRNAs or NC siRNAs for 48 h, using riboFECT^TM^ CP Transfection Kit (C10511-05, RiboBio, Guangzhou, China) in line with manufacturer's instructions. M0 macrophages were inoculated into 6-well plates at 5 × 10^5^ cells/well, and cultured overnight at 37°C in 5% CO_2_ incubator. Total RNA, protein and cell culture supernatant samples were all extracted or collected after siRNA transfection, prepared for use in the further analysis. The RT-qPCR analysis was applied in the validation of *YTHDF3* mRNA silencing efficiency. Additionally, positive control siRNA conjugated with Cy3 fluorophore (siT0000002-1-5, RiboBio, Guangzhou, China) was also used as the indication of successful transfection.

To further explore the role of *YTHDF3* in macrophage M1 polarization, induction of M1 polarization (fresh medium with 100 ng/mL LPS and 50 ng/mL IFN-γ, incubating for 24 h) was performed again after 48 h of *YTHDF3* siRNA transfection. Relevant samples were collected and stored for subsequent experiments.

### Enzyme-Linked Immunosorbent Assay (ELISA) for IL-12 Expression Levels

The expression levels of macrophage M1-phenotype marker IL-12 in cell culture supernatant were tested through ELISA. To begin with, cell culture media was centrifuged at 2,000 rpm for 20 min under 4°C, and then supernatant was obtained and ready for detection after the removal of impurities and cell debris. We used Mouse IL-12 ELISA Kit (D721111, Sangon Biotech, Shanghai, China) which employs the sandwich enzyme immunoassay technique for ELISA, following the manufacturer's protocol. After the formation of immune complex and its chromogenic reaction with TMB, the absorbance (OD) value of each sample was measured at 450 nm. The concentration of IL-12 in the sample was proportional to the OD value and could be calculated by quantitative method of standard curve.

### RNA Immunoprecipitation Sequencing (RIP-Seq) Analysis of *YTHDF3*

RIP experiments were performed using a Magna RIP RNA-binding Protein Immunoprecipitation Kit (Millipore, Billerica, MA, USA) in accordance with the manufacturer's instructions. First of all, nuclear protein was extracted from both AAA tissue and healthy aorta tissue for subsequent RIP-Seq. In brief, after coating with anti-*YTHDF3* antibody (sc-377119, Santa Cruz Biotechnology, Dallas, USA) or corresponding NC IgG antibody (Millipore, Billerica, MA, USA), cell lysates from AAA group and normal aorta group were independently used to incubate the coated magnetic beads overnight under 4°C. Later, the specific binding of RNA-protein complexes was treated in accordance with specific protocol for the two types of purified co-precipitated RNAs, and the RNA content was then analyzed by adopting NanoDrop 2000c. Furthermore, we eliminated rRNAs from the immune-precipitated RNAs. Later, we commissioned SEQHEALTH Ltd. Co. (Wuhan, China; Contract No. KC2019-D146) to conduct RIP-transcriptome sequencing analysis of the two groups of our immune-precipitated RNAs and the subsequent bioinformatics analysis. The RIP-Seq results were then uploaded to NCBI Sequence Read Archive (SRA) database. At last, the *YTHDF3*-binding genes in AAA tissue and healthy aorta tissue were compared and the AAA-specific genes bound to *YTHDF3* were identified.

The objective of RIP-Seq analysis of *YTHDF3* was to explore the potential function of *YTHDF3* by identifying the RNAs binding to *YTHDF3* protein in human AAA. Finally, the result of RIP-Seq analysis of *YTHDF3* was output and used for PPI network construction and the prediction of *YTHDF3* downstream target genes in AAA progression.

### Protein-Protein Interaction (PPI) Network Construction and Prediction of *YTHDF3* Downstream Target Genes in AAA Progression

2298 AAA-specific genes binding to *YTHDF3* were identified *via* RIP-Seq analysis. In order to dig the downstream target genes (DTGs) of *YTHDF3* that also play substantial roles in AAA progression, key intersection genes were yielded through the overlapping of these 2,298 genes, upregulated DEGs in AAA and the positive co-expression genes of *YTHDF3* in AAA.

The Search Tool for the Retrieval of Interacting Genes (STRING) database (https://www.string-db.org) was employed to construct a PPI network for key intersection genes. Thereafter, the PPI pairs were extracted using a combined score of >0.55. Subsequently, the *YTHDF3*-centric PPI network was visualized by Cytoscape 3.9.0 software (https://cytoscape.org/). According to the Maximal Clique Centrality (MCC) method and Degree method (Deg), CytoHubba was employed to calculate the MCC value as well as the degree of each protein node in the whole PPI network. Furthermore, MCODE, a plugin in Cytoscape, was utilized to analyze and visualize the sub-networks (highly interconnected clusters) in the PPI network. Lastly, nodes with the top 20 degrees or top 20 MCC values in the whole PPI network were defined as hub genes. These hub genes were also considered as DTGs of *YTHDF3* that might participate in the progression of AAA.

### Statistical Analysis

Statistical analysis was performed using SPSS 25.0 software (IBM Corp, Chicago, IL, USA) together with GraphPad Prism 8.0.1 software (GraphPad Software, San Diego, CA, USA) to evaluate differences among groups. Either the parametric Student's *t*-test for unpaired samples or the non-parametric Mann-Whitney U test was applied to analyze based on the distribution of variables. Comparisons among ≥3 groups were conducted using one-way ANOVA (with Brown-Forsythe test). For correlation analysis in our bioinformatics analysis section, correlations between different variables were analyzed through Pearson correlation analysis. A difference of *P value* <0.05 suggested statistical significance. All experiments were repeated in triplicates.

## Results

### Identification and Diagnostic Value Analysis of DEMRGs in AAA

**Figure 1:**
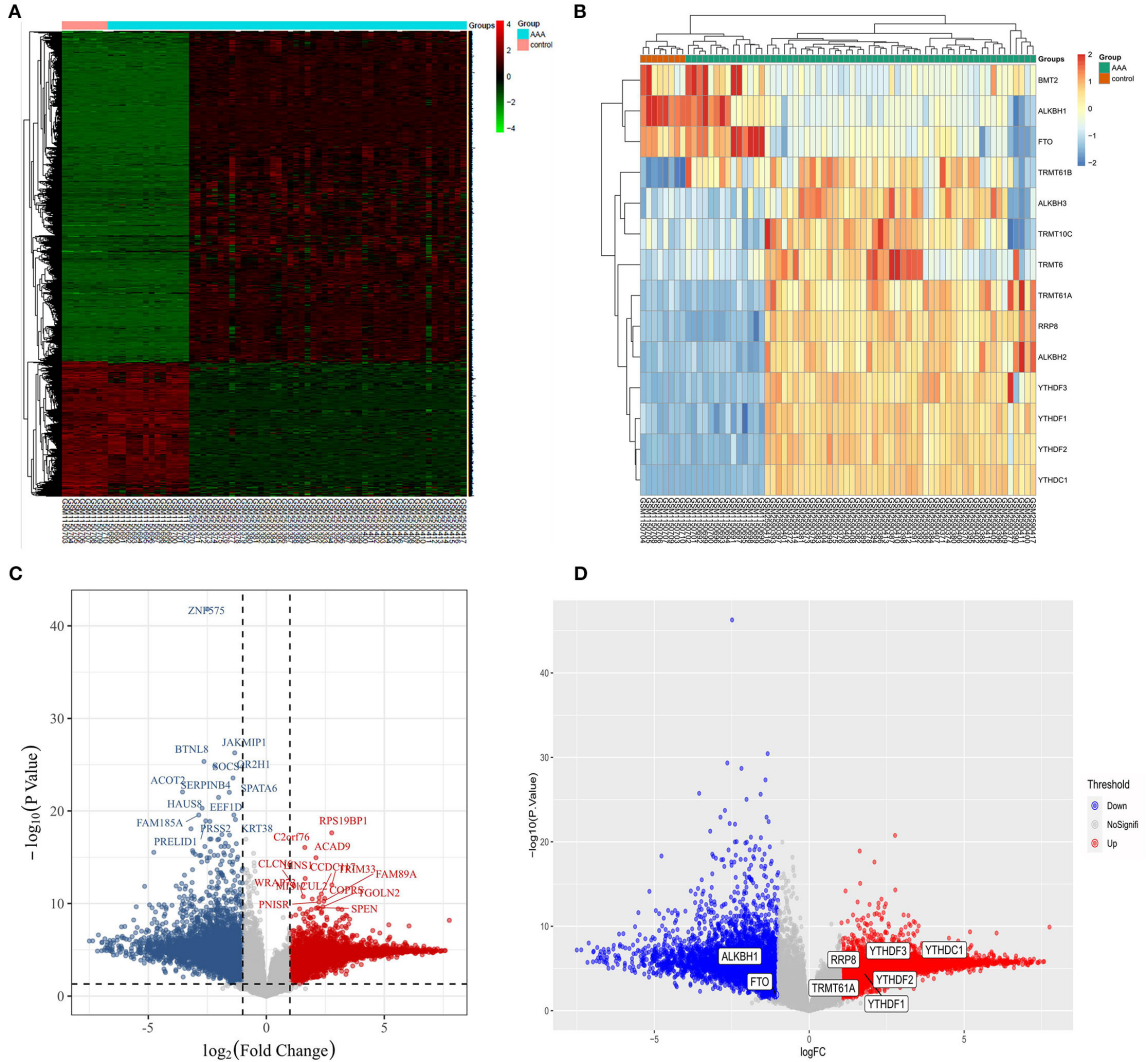
Screening of differentially expressed m1A regulatory genes in AAA. **(A)** Overall heat map of the differential expression analysis; **(B)** The expression pattern of 14 common m1A regulators in AAA and control samples; **(C)** The volcano plot of DEGs in AAA, red represents up-regulated genes and blue represents down-regulated; **(D)** The volcano plot showing 8 DEMRGs in AAA, red represents up-regulated genes and blue represents down-regulated genes.

**Figure 2:**
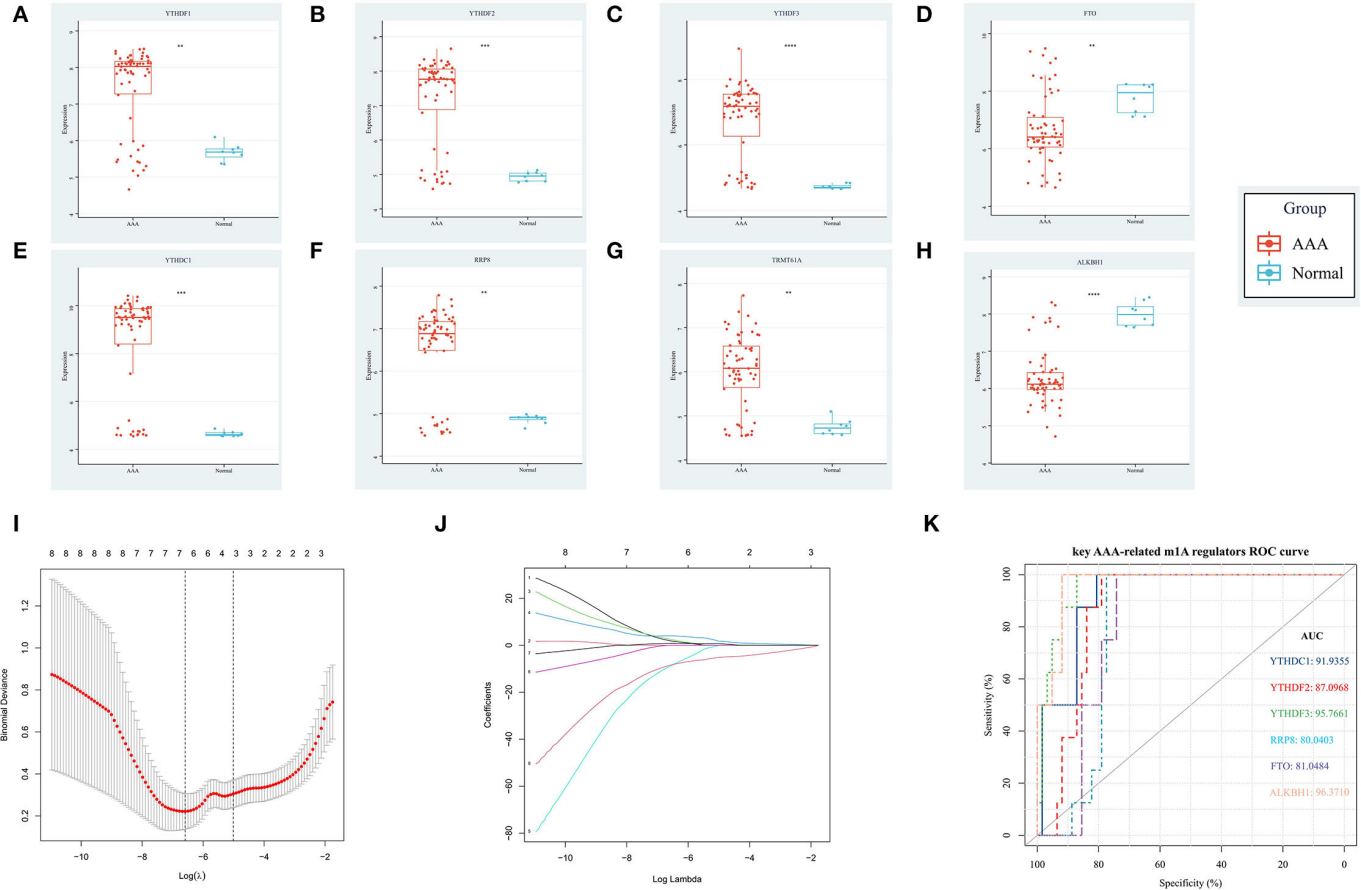
The differential expression level and diagnostic value of DEMRGs in AAA. **(A)** The up-regulating expression level of *YTHDF1*; **(B)** The up-regulating expression level of *YTHDF2*; **(C)** The up-regulating expression level of *YTHDF3*; **(D)** The down-regulating expression level of *FTO*; **(E)** The up-regulating expression level of *YTHDC1*; **(F)** The up-regulating expression level of *RRP8*; **(G)** The up-regulating expression level of *TRMT61A*; **(H)** The down-regulating expression level of *ALKBH1*; **(I)** The LASSO regression model established by 8 DEMRGs; **(J)** The coefficients plot of 8 DEMRGs in the LASSO model; **(K)** The ROC curves with AUC values of 6 key AAA-related m1A regulators.

Based on the combined dataset and the statistical threshold of |*log*_2_*FC*| ≥ 1 and adjusted *P* < 0.05, the differential expression analysis of genes between AAA samples and controls was conducted, as shown in the heat map (Figure 1A) and volcano plot of DEGs (Figure 1C). Later, the overlapping between DEGs and 14 common m1A regulators was carried out and 8 DEMRGs were identified through the analysis. The expression pattern of DEMRGs was shown in the mosaic plot (Figure 1B) and volcano plot (Figure 1D). 8 DEMRGs, containing *YTHDC1, YTHDF1-3, RRP8, TRMT61A, FTO*, and *ALKBH1*, were distinctly displayed in Table 2. The significantly differential expression of DEMRGs between AAA groups and control groups was illustrated in box plots (Figures 2A–H).

**Table 2:** Identification of 8 AAA-related m1A regulators.

**m1A regulator**	**Function**	***Log_**2**_FC***	**Adj. *P***	**Differential expression**
***YTHDC1***	m1A “reader”	3.849	<0.001	UP
***YTHDF2***	m1A “reader”	2.244	<0.001	UP
***YTHDF3***	m1A “reader”	2.034	<0.001	UP
***YTHDF1***	m1A “reader”	1.779	<0.001	UP
***RRP8***	m1A “writer”	1.619	<0.001	UP
***TRMT61A***	m1A “writer”	1.252	<0.001	UP
***FTO***	m1A “eraser”	−1.080	<0.050	DOWN
***ALKBH1***	m1A “eraser”	−1.722	<0.001	DOWN

*m1A, N1-Methyladenosine; FC, Fold Change; Adj. P, Adjusted P value*.

The expression profile of the 8 DEMRGs was extracted and used to establish the LASSO model (Figures 2I,J). Then, 6 genes, including *YTHDC1, YTHDF2-3, RRP8, FTO*, and *ALKBH1* were identified *via* the LASSO binomial regression analysis based on the value of λ.min = 0.001374812 and therefore were qualified for the further ROC curve analysis. Thereafter, ROC curve analysis demonstrated the AUC value of *ALKBH1* (96.37), *YTHDF3* (95.77%), *YTHDC1* (91.94%), *YTHDF2* (87.10%), *FTO* (81.05%), and *RRP8* (80.04%), indicating *ALKBH1, YTHDF3* and *YTHDC1* may serve as potential biomarkers of AAA with high diagnostic values (Figure 2K). To be specific, *YTHDF3* has the highest diagnosis value (with AUC > 95%) among all the up-regulated DEMRGs, which implies the significance of studying the potential function of *YTHDF3* in our subsequent analysis.

### Functional Enrichment and Pathway Analysis of Co-expressed Genes of DEMRGs

After the co-expression analysis of all the DEMRGs, a union set of 7,237 co-expressed genes were yielded and then input into the gene pool which was used for GO and KEGG enrichment analysis (Supplementary Material 1). As indicated by GO analysis, these co-expressed genes were mainly involved in BPs, including RNA catabolic process, neutrophil-mediated immunity, protein targeting, regulation of cellular amide metabolic process and regulation of cell cycle phase transition. In terms of CCs, these genes were mostly enriched in the mitochondrial inner membrane, mitochondrial matrix, cell-substrate junction and focal adhesion. With regard to MFs, these genes were mainly enriched in transcription co-regulator activity, GTPase regulator activity, cadherin binding and ubiquitin protein ligase binding, etc. Additionally, the KEGG pathway enrichment results showed that these genes were significantly enriched in pathways participated in chemical carcinogenesis-reactive oxygen species (ROS), endocytosis and protein processing in endoplasmic reticulum (ER). The outcome of functional enrichment and pathway analysis of co-expressed genes was presented in Supplementary Material 2 and Table 3.

**Table 3:** GO and KEGG enrichment terms of co-expressed genes of DEMRGs.

**Category**	**Term**	**Count**	**Adj. *P value***
GO~BP	GO:0006401~RNA catabolic process	223	1.31E-24
	GO:0042119~neutrophil activation	223	1.98E-12
	GO:0002446~neutrophil mediated immunity	219	1.85E-11
	GO:0043312~neutrophil degranulation	218	1.67E-12
	GO:0002283~neutrophil activation involved in immune response	218	2.80E-12
	GO:0010498~proteasomal protein catabolic process	214	1.20E-11
	GO:0006605~protein targeting	204	4.02E-13
	GO:0008380~RNA splicing	202	1.39E-08
	GO:0006402~mRNA catabolic process	201	7.84E-22
	GO:0034248~regulation of cellular amide metabolic process	200	3.50E-07
	GO:0022613~ribonucleoprotein complex biogenesis	194	1.99E-07
	GO:0034660~ncRNA metabolic process	192	1.18E-05
	GO:0009896~positive regulation of catabolic process	188	8.08E-08
	GO:0043161~proteasome-mediated ubiquitin-dependent protein catabolic process	187	8.80E-10
	GO:1901987~regulation of cell cycle phase transition	187	1.39E-05
GO~CC	GO:0005743~mitochondrial inner membrane	218	3.83E-12
	GO:0005759~mitochondrial matrix	214	1.29E-12
	GO:0030055~cell-substrate junction	204	8.87E-16
	GO:0005925~focal adhesion	203	3.04E-16
	GO:0005774~vacuolar membrane	189	4.20E-10
	GO:0016607~nuclear speck	179	2.90E-09
	GO:0005635~nuclear envelope	165	0.004652
	GO:0005765~lysosomal membrane	163	3.65E-08
	GO:0098852~lytic vacuole membrane	163	3.65E-08
	GO:0005769~early endosome	156	2.47E-06
	GO:0005819~spindle	152	2.42E-05
	GO:0005667~transcription regulator complex	149	0.003521
	GO:0031252~cell leading edge	149	0.004348
	GO:0098798~mitochondrial protein-containing complex	145	1.83E-16
	GO:0031300~intrinsic component of organelle membrane	143	0.012147
GO~MF	GO:0003712~transcription coregulator activity	210	2.14E-08
GO~MF	GO:0030695~GTPase regulator activity	178	0.005691
	GO:0045296~cadherin binding	156	1.99E-08
	GO:0140098~catalytic activity, acting on RNA	151	0.004986
	GO:0140297~DNA-binding transcription factor binding	146	0.009294
	GO:0044389~ubiquitin-like protein ligase binding	136	2.57E-05
	GO:0031625~ubiquitin protein ligase binding	126	0.000154
	GO:0061629~RNA polymerase II-specific DNA-binding transcription factor binding	116	0.00045
	GO:0003713~transcription coactivator activity	113	0.000179
	GO:0051020~GTPase binding	110	2.21E-07
	GO:0003735~structural constituent of ribosome	93	2.54E-07
	GO:0031267~small GTPase binding	91	2.33E-05
	GO:0008022~protein C-terminus binding	85	0.000367
	GO:0003714~transcription corepressor activity	83	0.000613
	GO:0043021~ribonucleoprotein complex binding	79	4.47E-09
KEGG	hsa05022~Pathways of neurodegeneration - multiple diseases	202	2.45E-05
	hsa05014~Amyotrophic lateral sclerosis	169	6.75E-07
	hsa05010~Alzheimer's disease	152	0.009071
	hsa05016~Huntington disease	137	4.67E-05
	hsa05012~Parkinson's disease	126	5.68E-06
	hsa05020~Prion disease	125	3.76E-05
	hsa05132~Salmonella infection	109	0.000898
	hsa04714~Thermogenesis	107	9.05E-05
	hsa04144~Endocytosis	105	0.007389
	hsa05171~Coronavirus disease - COVID-19	104	0.000458
	hsa05131~Shigellosis	103	0.008748
	hsa05208~Chemical carcinogenesis - reactive oxygen species	100	0.000606
	hsa05203~Viral carcinogenesis	85	0.021432
	hsa05415~Diabetic cardiomyopathy	84	0.027233
	hsa04141~Protein processing in endoplasmic reticulum	83	9.30E-05

*GO, Gene Ontology; BP, biological processes; CC, cellular components; MF, molecular functions; KEGG, Kyoto Encyclopedia of Genes and Genomes; Adj. P value, Adjusted P value; DEMRGs, differentially expressed m1A regulatory genes*.

### Results of Immune Cell Infiltration and Its Correlation With DEMRGs in AAA

**Figure 3:**
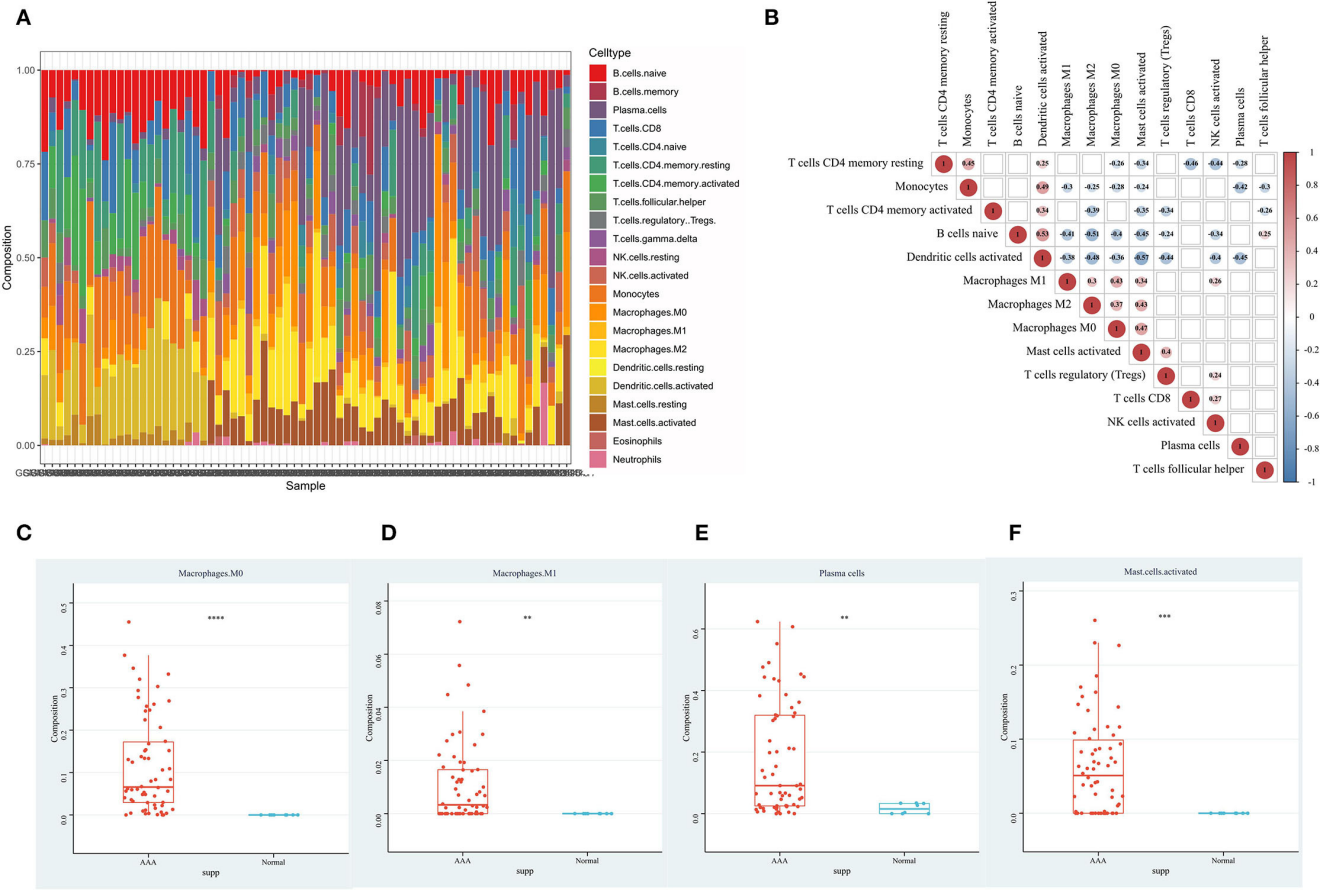
The immune infiltration landscape of AAA. **(A)** The composition of 22 types of immune cells in each sample; **(B)** The correlation among immune cells in AAA samples, with red representing positive correlation and blue representing negative correlation. Correlation results that were not statistically significant were shown as blank; **(C)** The content of M0 macrophages in normal aortic samples and AAA samples; **(D)** The content of M1 macrophages in normal aortic samples and AAA samples; **(E)** The content of plasma cells in normal aortic samples and AAA samples; **(F)** The content of activated mast cells in normal aortic samples and AAA samples.

**Figure 4:**
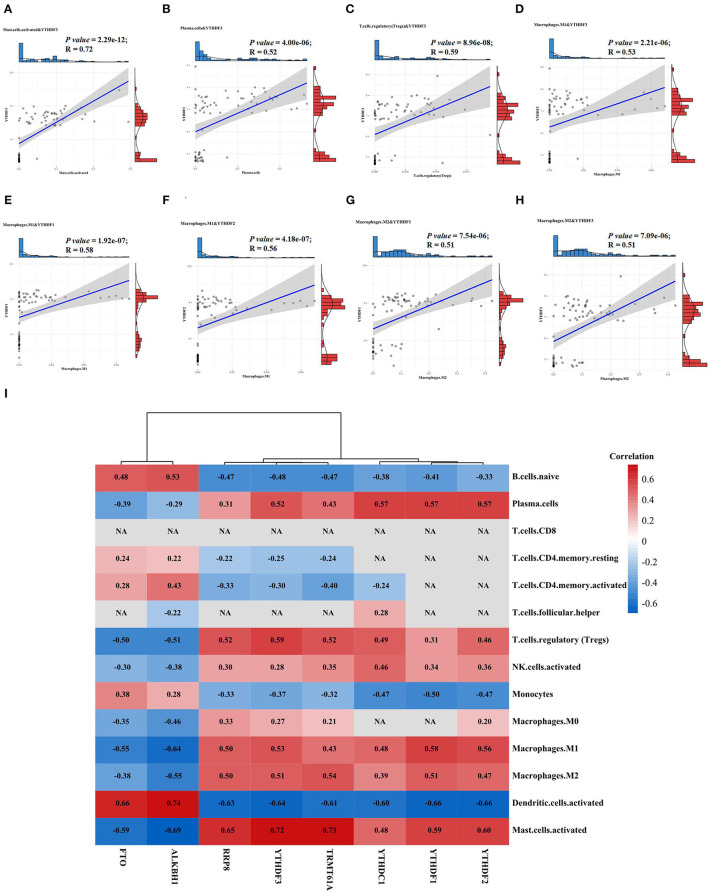
The relationships between DEMRGs and immune cells. **(A)** Scatter plot of the correlation between *YTHDF3* and activated mast cells; **(B)** Scatter plot of the correlation between *YTHDF3* and plasma cells; **(C)** Scatter plot of the correlation between *YTHDF3* and regulatory T cells (Tregs); **(D)** Scatter plot of the correlation between *YTHDF3* and M1 macrophages; **(E)** Scatter plot of the correlation between *YTHDF1* and M1 macrophages; **(F)** Scatter plot of the correlation between *YTHDF2* and M1 macrophages; **(G)** Scatter plot of the correlation between *YTHDF1* and M2 macrophages; **(H)** Scatter plot of the correlation between *YTHDF3* and M2 macrophages; **(I)** Correlation heat map of the relationships between DEMRGs and immune cells, with red representing positive correlation, blue representing negative correlation and NA representing no statistical significance.

Through the immune cell infiltration analysis, the relative contents of 22 types of immune cells in 62 AAA samples and 8 normal samples were calculated by the CIBERSORT algorithm (Figure 3A). The landscape of immune infiltration in AAA tissues indicated the significantly higher relative proportions of M0 macrophages, M1 macrophages, plasma cells and activated mast cells in AAA than normal tissues (Figures 3C–F). Moreover, after filtering out 8 immune cell types with extremely low proportions, the Pearson correlation analysis was performed to evaluate the correlation among immune cells as well as the correlation between DEMRGs and immune cells in all samples (Figures 3B, 4I). For example, activated dendritic cells were markedly positively correlated with naive B cells (*R* = 0.53) and monocytes (*R* = 0.49); M0 macrophages were significantly correlated with activated mast cells (*R* = 0.47).

As for the relationship between DEMRGs and immune cells, we found that *YTHDF3* had the most significantly positive correlation with various immune cells, such as activated mast cells (*R* = 0.73), Treg cells (*R* = 0.59), plasma cells (*R* = 0.52), M1 macrophages (*R* = 0.53), and M2 macrophages (*R* = 0.51). Among these immune cells, macrophages served as the appropriate research subject for the further analysis of *YTHDF3*'s regulatory effect, due to its known important role in the formation and progression of AAA. Besides, some of the typical correlations between DEMRGs and immune cells were presented in scatter diagrams (Figures 4A–H).

### The mRNA Expression Levels of Up-Regulated DEMRGs in AAA Samples

**Figure 5:**
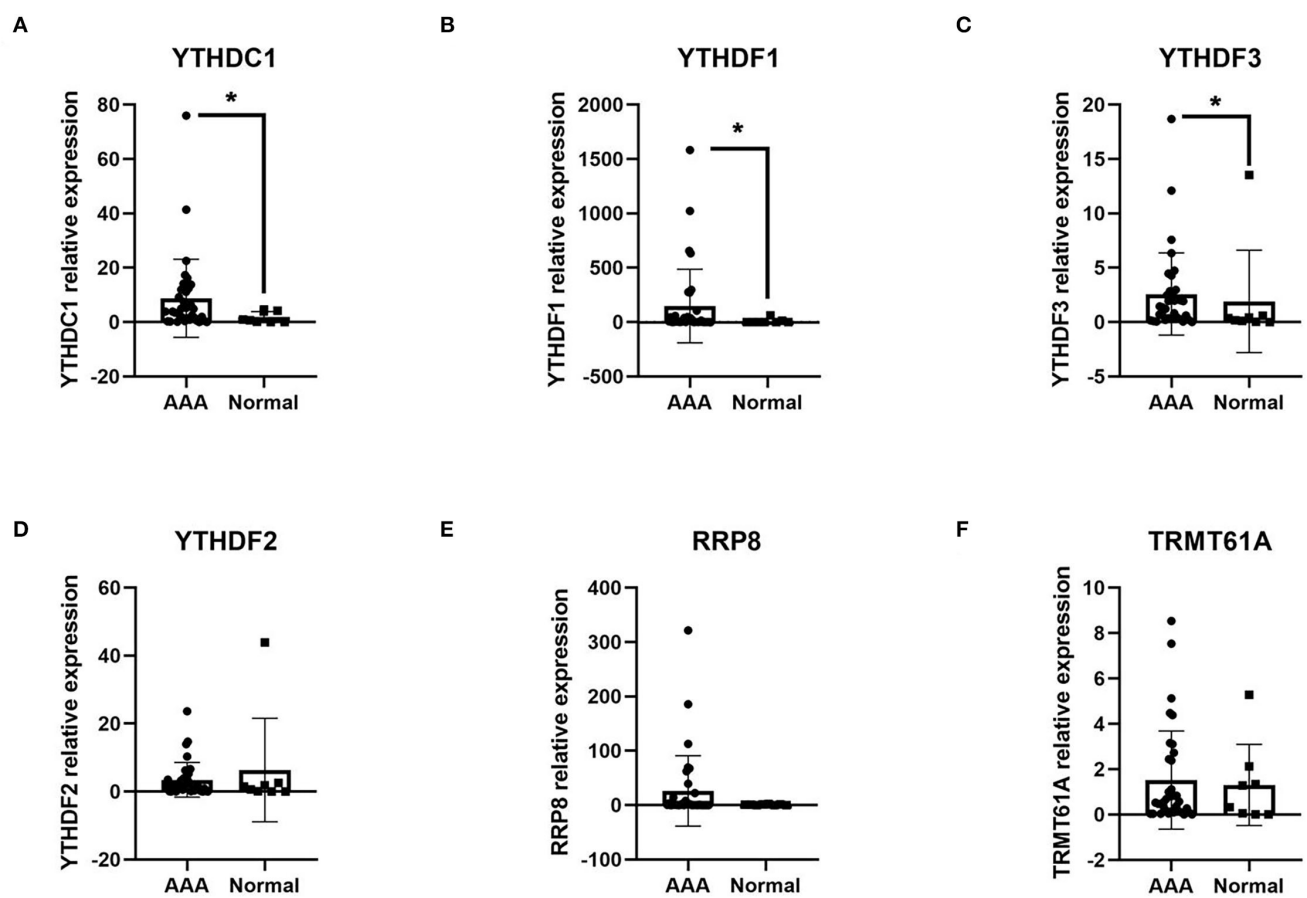
The validation of the expression of DEMRGs at mRNA level in AAA tissue samples compared with the healthy control aortic samples, analyzed by RT-qPCR. **(A)** The relative expression of *YTHDC1* in two groups; **(B)** The relative expression of *YTHDF1* in two groups; **(C)** The relative expression of *YTHDF3* in two groups; **(D)** The relative expression of *YTHDF2* in two groups; **(E)** The relative expression of *RRP8* in two groups; **(F)** The relative expression of *TRMT61A* in two groups. *P < 0.05.

Thereafter, 6 up-regulated DEMRGs, including *YTHDC1, YTHDF1-3, RRP8*, and *TRMT61A*, were validated by RT-qPCR analysis for their relative expression at mRNA level (Figure 5). In our current study, the mRNA expressions of *YTHDC1, YTHDF1*, and *YTHDF3* were observed up-regulating in the AAA tissue samples relative to normal tissues (FC = 4.606, *P* = 0.043; FC = 26.927, *P value* = 0.031; FC = 5.426, *P* = 0.043). Differences in *YTHDF2, RRP8*, and *TRMT61A* at mRNA levels were not significant (*P* = 0.364, *P* = 0.567, and *P* = 0.775).

Hence, 3 AAA-Related m1A regulators named *YTHDC1, YTHDF1*, and *YTHDF3* were validated as up-regulated DEMRGs in our AAA samples.

### The Protein Expression and Cellular Localization of *YTHDF3* in AAA Tissues

**Figure 6:**
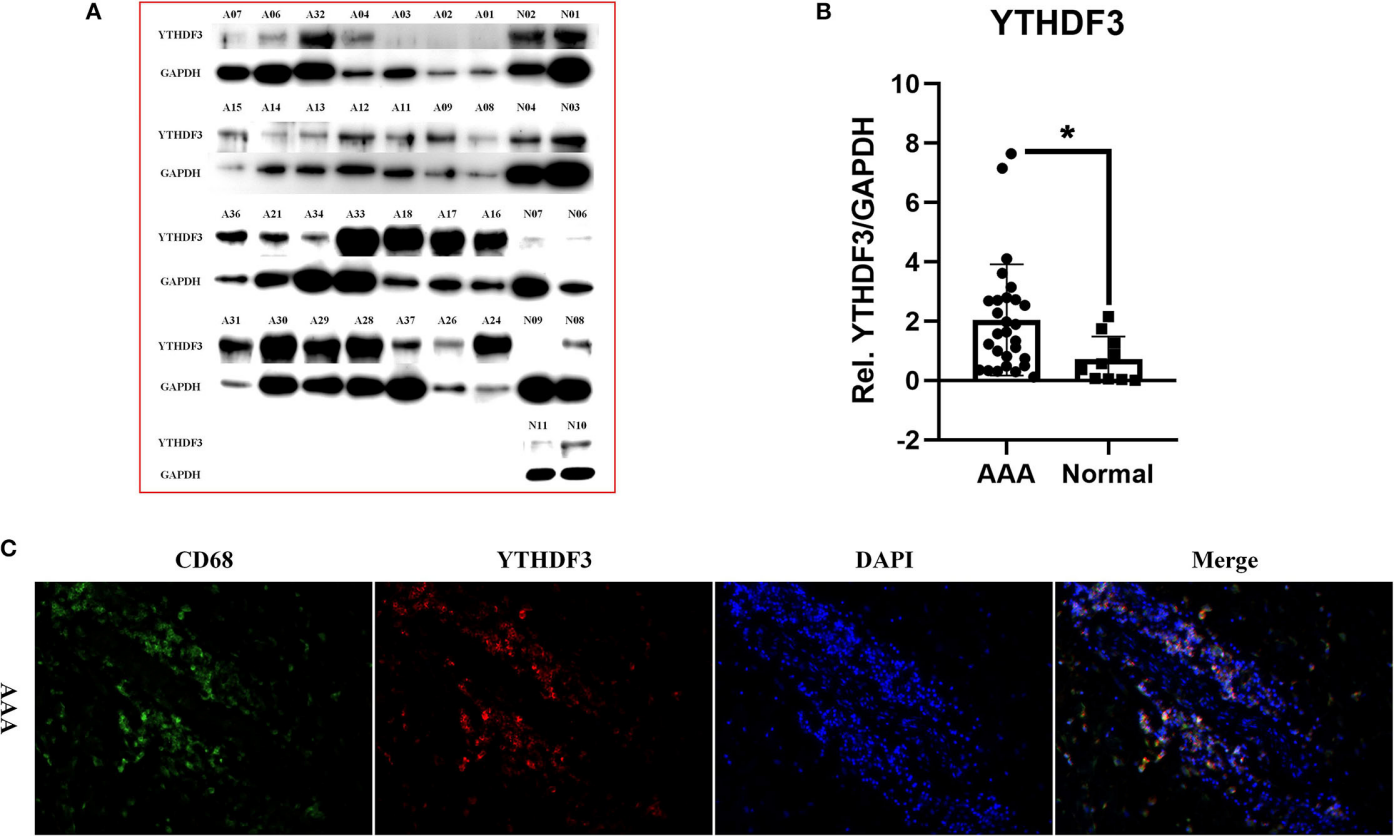
The expression of *YTHDF3* at protein level and its cellular localization in AAA tissues. **(A)** The images of Western Blot staining for *YTHDF3* and *GAPDH* in each sample; **(B)** The box plot showing the relative expression of *YTHDF3* (normalized to the signal intensity of *GAPDH*) in AAA and normal aortic samples; **(C)** The photographs of immunofluorescence for human AAA sections stained with *CD68, YTHDF3* and 4', 6-diamidino-2-phenylindole (DAPI). *P < 0.05.

We selectively performed Western Blot analysis for *YTHDF3* in AAA and normal aorta tissues, because of its potential high diagnostic value, its strong positive correlation with immune infiltration and its up-regulating expression at mRNA level in human AAAs. Figures 6A,B displayed the blot images of *YTHDF3* and its relative expression levels measured through Western blotting in all samples. The protein expression level of *YTHDF3* in AAA tissue samples was significantly increased compared with healthy control aortic tissue samples (FC = 3.475, *P* = 0.012), in accordance with the RT-qPCR result.

Later, to further validate the relationship between *YTHDF3* and macrophages, the AAA wall co-localization of *YTHDF3* with macrophage surface marker *CD68* was examined using IF double staining analysis (Figure 6C). The IF result showed a significant infiltration of macrophages in AAA adventitia. Here, we could also clearly observe that *YTHDF3* and *CD68* were co-expressed in the one cell in AAA adventitia, which indicated the potential significance of studying the role of *YTHDF3* in macrophage functions.

### The Up-Regulation Relative Expression of *YTHDF3* Associated With Macrophage M1 Polarization

**Figure 7:**
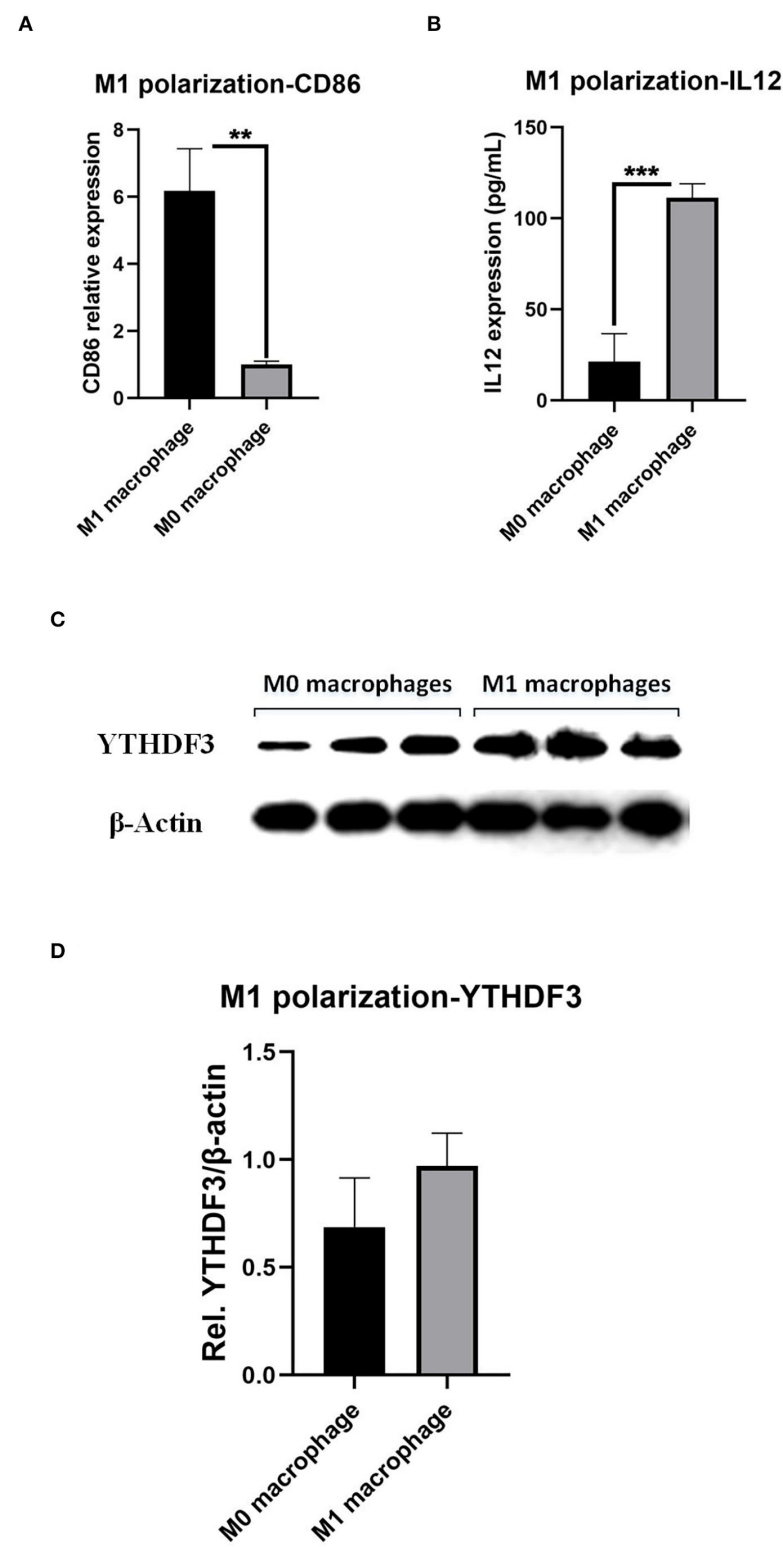
The induction of macrophages M1 polarization and the determination of *YTHDF3* expression in M0 and M1 macrophages. **(A)** The expression of M1 phenotype marker, *CD86*, in M0 macrophages and LPS/IFN-γ induced M1 macrophages, measured by RT-qPCR; **(B)** The expression of M1 phenotype marker, IL12, in M0 macrophages and LPS/IFN-γ induced M1 macrophages, measured by ELISA; **(C)** The images of Western Blot staining for *YTHDF3* and β*-Actin*, in M0 and LPS/IFN-γ induced M1 macrophages; **(D)** The relative expression of *YTHDF3* (normalized to the signal intensity of β*-Actin*) in M0 and LPS/IFN-γ induced M1 macrophages. LPS, lipopolysaccharide; IFN-γ, interferon-γ. **P < 0.01, ***P < 0.001.

Since the immune cell infiltration analysis together with the IF experiment illustrated the strong correlation between *YTHDF3* and M1/M2 macrophages, we started to focus on the association between *YTHDF3* and macrophage pro-inflammatory M1 polarization, which was involved in the immune-driven destruction of the aortic wall. Firstly, after the LPS/IFN-γ stimulus, the expression of M1 phenotype marker *CD86* and IL12 significantly increased, indicating an ideal M1 macrophages experimental model (Figures 7A,B). Thereafter, the relative expression of *YTHDF3* in M0 macrophage and M1 macrophage was determined by Western Blot analysis and the result showed an up-regulating tendency of *YTHDF3* in M1 macrophage than M0 macrophage (FC = 1.412) but with the *P* = 0.149 (Figures 7C,D).

### The Role of *YTHDF3* in the M1/M2 Phenotype Transition of M0 Macrophage

**Figure 8:**
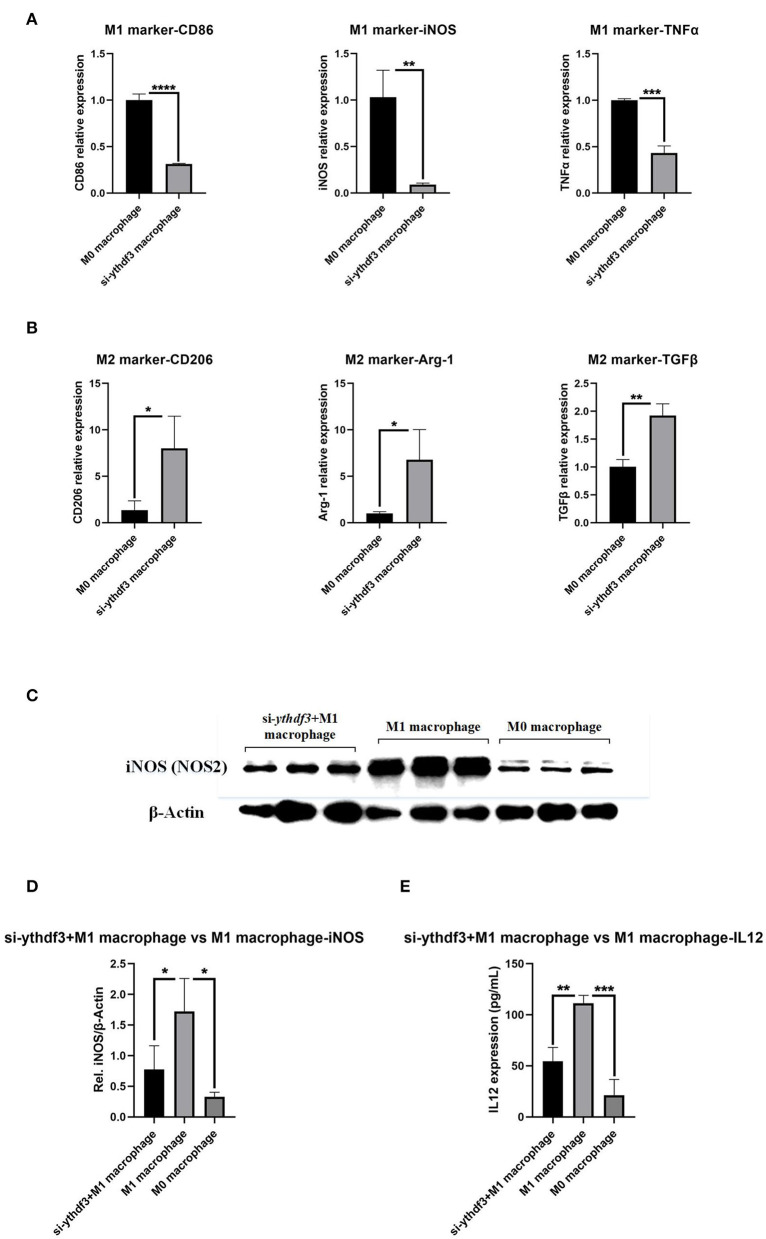
The role of *YTHDF3* in the M1/M2 polarization of M0 macrophages. *YTHDF3*; **(A)** The relative expression of M1 phenotype markers (*CD86, iNOS* and *TNF*α) in M0 macrophages and si-*ythdf3* macrophages, analyzed by RT-qPCR; **(B)** The relative expression of M2 phenotype markers (*CD206, Arg-1* and *TGF*β) in M0 macrophages and si-*ythdf3* macrophages, analyzed by RT-qPCR; **(C)** The images of Western Blot staining for *iNOS* and β*-Actin*, in M0 macrophages, LPS/IFN-γ induced M1 macrophages and si-*ythdf3*+M1 polarization macrophages; **(D)** The relative expression of *iNOS* (normalized to the signal intensity of β*-Actin*) in M0 macrophages, LPS/IFN-γ induced M1 macrophages and si-*ythdf3*+M1 polarization macrophages; **(E)** The expression of IL12 in M0 macrophages, LPS/IFN-γ induced M1 macrophages and si-*ythdf3*+M1 polarization macrophages, measured by ELISA. *P < 0.05; **P < 0.01; ***P < 0.001; ****P < 0.0001; *iNOS*, inducible nitric oxide synthase; *TNF*α, tumor necrosis factor-α; *Arg-1*, arginine-1; *TGF*β, transforming growth factor-β.

For the aim of further exploring the mechanism underlying the regulation of *YTHDF3* in macrophage M1/M2 polarization, siRNA transfection experiment was performed to specifically knock down the expression of *YTHDF3* in RAW264.7 cells. The RT-qPCR result of *YTHDF3* indicated an ideal *ythdf3* knockdown efficiency (FC = 0.199, *P* < 0.0001, Supplementary Material 3). Later, the RT-qPCR analysis was conducted to analyze the relative expression of M1 phenotype markers (*CD86, iNOS, TNF*α) and M2 phenotype markers (*CD206, Arg-1, TGF*β). The results demonstrated that siRNA knockdown of *YTHDF3* inhibited macrophages M1 polarization and at the same time promoted macrophages M2 polarization (Figures 8A,B).

### *YTHDF3* Knockdown Impaired LPS/IFN-γ Induced Macrophage M1 Polarization

For the *YTHDF3* knockdown group, subsequent induction of macrophage M1 polarization was carried out and the expression of Inducible Nitric Oxide Synthase (*iNOS, NOS2*) and Interleukin 12 (IL12), two of the biomarkers and functional products of M1 macrophages, was tested through Western Blot and ELISA. The comparisons were performed among si-*ythdf3*+M1 polarization macrophage group, M1 macrophage group and M0 macrophage group. The result showed that *YTHDF3* knockdown significantly impaired LPS/IFN-γ induced macrophage M1 polarization, and meanwhile attenuated the secretion of inflammatory factor IL12, significantly reversing the M0 to M1 polarization of macrophages (Figures 8C,E).

### The RIP-Seq Results and the Prediction of *YTHDF3* Downstream Targets Participating in AAA Progression

**Figure 9:**
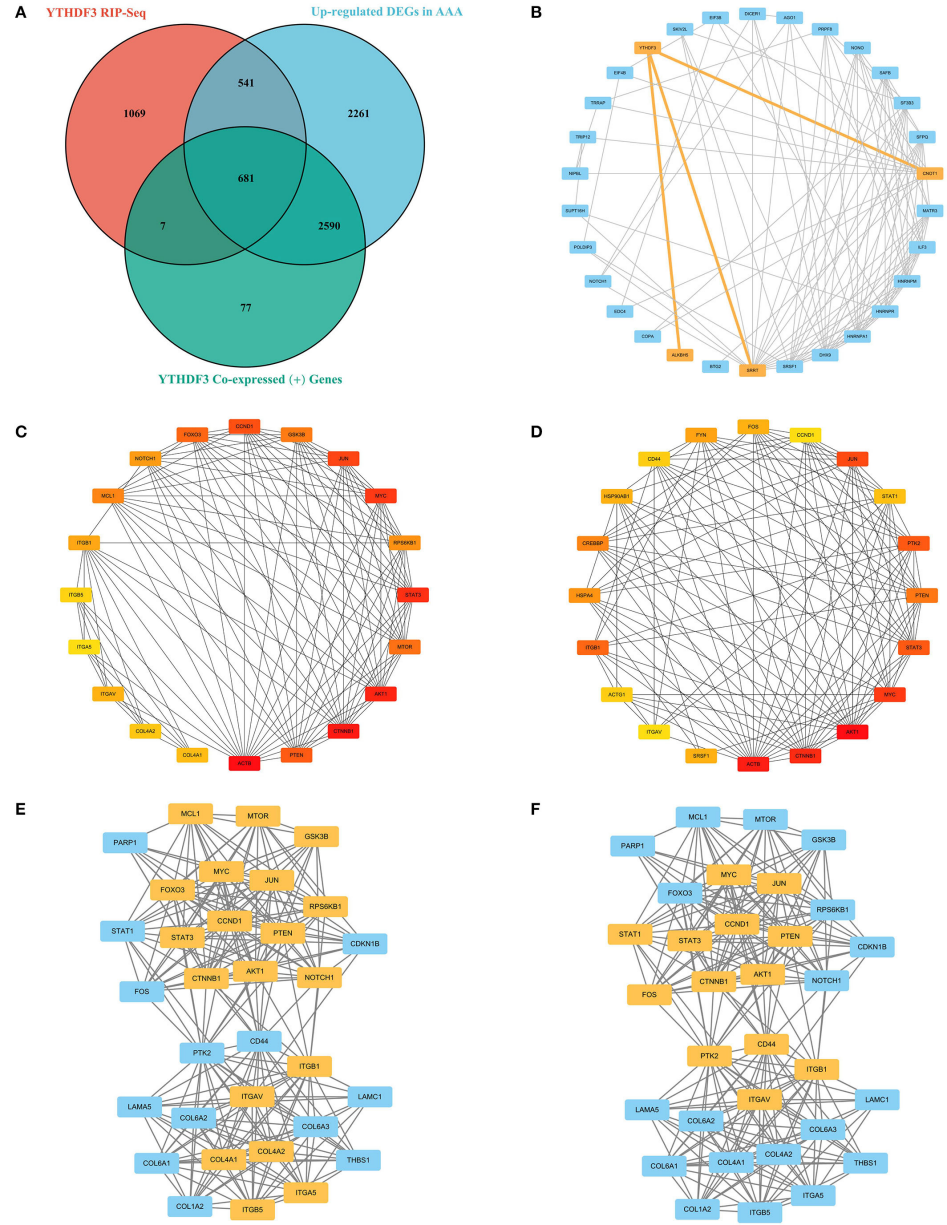
The construction of PPI network based on the RIP-Seq result and the prediction of *YTHDF3* downstream targets participating in AAA progression. **(A)** The Venn diagram showing the overlapping of the AAA-specific *YTHDF3*-binding genes*YTHDF3, YTHDF3* co-expressed (+) genes and up-regulated DEGs in AAA. **(B)** The two-level PPI network centered on *YTHDF3*; **(C)** The interactions among proteins with the top 20 MCC values in the whole PPI network. Red represents higher MCC values and yellow represents lower MCC values; **(D)** The interactions among proteins with the top 20 node degrees in the whole PPI network. Red represents higher node degrees and yellow represents lower node degrees; **(E)** The sub-network with the highest cluster score in the whole PPI network. Hub genes (identified by MCC method) existing in this sub-network were marked yellow; **(F)** The sub-network with the highest cluster score in the whole PPI network. Hub genes (identified by Degree method) existing in this sub-network were marked yellow. MCC, Maximal Clique Centrality.

First of all, the RIP-Seq result of *YTHDF3* in AAA tissue and healthy control sample was independently uploaded to the dataset SRX9734810 and SRX9734811, respectively, in NCBI SRA database. Afterwards, 2,298 AAA-specific *YTHDF3*-binding genes were yielded *via* the comparison with control group (Supplementary Material 4). Then, the overlapping of AAA-specific *YTHDF3*-binding genes*YTHDF3*, up-regulated DEGs in AAA and positively co-expressed genes of *YTHDF3* in AAA was conducted to obtain an intersection of 681 genes (Figure 9A). In addition, these 681 key intersection genes and *YTHDF3* were input into the STRING database to construct a PPI network (Supplementary Material 5). The two-level PPI network centered on *YTHDF3* was visualized by Cytoscape (Figure 9B). Thereafter, hub genes with top 20 MCC values or node degrees were identified and visualized in circular networks by CytoHubba (Figures 9C,D and Tables 4, 5). After the combination of two hub gene lists, the final 30 hub genes were predicted as *YTHDF3* downstream target genes (DTGs) participating in AAA progression. At last, MCODE plugin in Cytoscape was used to perform sub-network analysis of the whole PPI network. The sub-network with the highest cluster score was displayed in Figures 9E,F and hub genes identified by MCC method and Degree method were marked yellow in the diagram, respectively.

**Table 4:** Hub Genes with the Top 20 MCC values in the PPI network.

**Rank**	**Hub gene**	**MCC value**
1	*ACTB*	6.17E+08
2	*AKT1*	6.16E+08
3	*CTNNB1*	6.16E+08
4	*MYC*	6.16E+08
5	*JUN*	6.16E+08
6	*STAT3*	6.16E+08
7	*CCND1*	6.15E+08
8	*PTEN*	6.11E+08
9	*FOXO3*	6.10E+08
10	*MTOR*	5.63E+08
11	*GSK3B*	5.23E+08
12	*MCL1*	4.87E+08
13	*RPS6KB1*	4.83E+08
14	*NOTCH1*	1.21E+08
15	*ITGB1*	8.94E+07
16	*ITGAV*	8.93E+07
17	*COL4A2*	8.87E+07
18	*COL4A1*	8.87E+07
19	*ITGB5*	8.86E+07
20	*ITGA5*	8.84E+07

*PPI, Protein-Protein Interaction; MCC, Maximal Clique Centrality*.

**Table 5:** Hub genes with the Top 20 degrees in the PPI network.

**Rank**	**Hub gene**	**Node degree**
1	*AKT1*	102
2	*ACTB*	101
3	*CTNNB1*	91
4	*MYC*	75
5	*JUN*	64
6	*STAT3*	57
7	*PTK2*	57
8	*ITGB1*	56
9	*PTEN*	52
10	*CREBBP*	51
11	*HSPA4*	46
12	*FYN*	44
13	*FOS*	43
14	*SRSF1*	43
15	*HSP90AB1*	43
16	*STAT1*	42
17	*CD44*	41
18	*ACTG1*	41
19	*CCND1*	40
20	*ITGAV*	40

*PPI, Protein-Protein Interaction*.

## Discussion

Abdominal aortic aneurysm (AAA) is a severe vascular disease that can carry extremely high mortality rates (8). Currently, there remains a paucity of medical therapies to help mitigate the rate of aneurysm growth and prevent AAA rupture (29). However, the consistent research progress on the roles of epigenetic regulation, especially RNA methylation in AAA pathogenesis, has opened up new therapeutic possibilities of AAA. For instance, Zhong et al. pointed out that *METTL3* induces AAA development by modulating N6-Methyladenosine (m6A)-dependent primary *miR34a* processing (30). Similar to m6A modification, N1-Methyladenosine (m1A) is a typical type of RNA methylation, executing its biological functions through post-transcriptional regulation. Besides its role in tumorigenesis and many other biological processes (31), characteristic m1A modification and regulation have been reported in the pathophysiology of various cardiovascular diseases (CVDs) (17, 32, 33). Hence, it is of great significance to explore the role of m1A regulation in AAA occurrence and development.

First of all, in the bioinformatics analysis part of this study, we identified 8 DEMRGs and their co-expressed genes, followed by the GO/KEGG enrichment analysis. The result indicated that these m1A regulators and their related genes were significantly associated with biological processes like neutrophil activation/neutrophil-mediated immunity, ncRNA metabolic process and regulation of cell cycle phase transition. Scientists have found that the number of neutrophils and neutrophil-lymphocyte ratio is not only correlated with in-hospital mortality in symptomatic unruptured AAA patients (34), but also the overall mortality after endovascular AAA repair (35). Therefore, m1A regulators and their related genes probably influence the prognosis of AAA *via* regulating the immune infiltration in AAA. In addition, the ncRNA regulation of programmed cell death in VSMCs is gaining increasing attention nowadays. Le et al. and Wang et al. illustrated that long non-coding RNA (lncRNA) *GAS5* is able to regulate human aortic smooth muscle cells (HASMCs) apoptosis and proliferation through the *miR-185-5p/ADCY7miR-185-5p/ADCY7* axis, thus promoting AAA formation (36, 37). From this perspective, our study implied that the interplay between m1A regulation and non-coding RNAs may cause the programmed cell death of VSMCs by modulating the cell cycle phase transition. Furthermore, enrichment analysis showed that DEMRGs and their related genes were highly expressed in the cellular components including cell-substrate junction and focal adhesion, then executive molecular function like cadherin binding and participate in cell communication. A variety of studies have proved that the intercellular interaction and cell communication among VSMCs, immune cells and human aortic endothelial cells (hAECs) are strongly associated with AAA progression (38, 39). The KEGG pathway enrichment indicated a significant involvement of m1A regulators and their regulatory genes in pathways of reactive oxygen species (ROS), which is considered as a key factor of vascular oxidative stress, contributing to AAA pathogenesis (40, 41).

Secondly, RT-qPCR analysis verified that three of the m1A regulators, named *YTHDC1, YTHDF1* and *YTHDF3*, were up-regulated in AAAs at mRNA level. *YTHDC1, YTHDF1* and *YTHDF3*, both containing the YTH (YT521-B homology) domain, belong to the “readers” family of m1A methylation, functioning as methylation recognition enzymes that can read methylation sites and corporate with methylases (proteins of “writers” family) (17). *YTHDC1* may participate in the RNA alternative splicing and affect the export of methylated mRNA from the nucleus to the cytoplasm, acting as the biomarker of prostate cancer and colon adenocarcinoma (42). *YTHDF1* and *YTHDF3* have all been identified as translation regulators, which are responsible for interacting with different mRNA targets (43). Besides, while *YTHDF1* functions in translation regulation, its partner, *YTHDF3*, can serve as a hub for fine-tuning the RNA accessibility of *YTHDF1*, significantly promoting the translation of its target mRNAs by interacting with *YTHDF1* (42). Other reports also demonstrated that cytoplasmic *YTHDF3* promotes mRNA translation in synergy with *YTHDF1*, meanwhile affecting methylated mRNA decay mediated through *YTHDF2* and serves as an initiation factor of protein synthesis from circRNAs in human cells (44–46). For the potential roles of *YTHDF3* in cardiovascular diseases, genome-wide association studies (GWAS) showed that SNP rs4739066 on *YTHDF3* is significantly associated with myocardial infarction (MI) and Shi et al. also found that *YTHDF3* is dramatically differentially expressed in MI tissues compared with normal controls (47, 48).

Recent studies have frequently associated m1A regulator-mediated modification patterns with tumor microenvironment-infiltrating immune cells or other immunological characteristics in cancers like Colon Cancer and Ovarian Cancer (19, 49). According to the first part of our bioinformatics analysis, we also concluded that m1A regulators and their related genes were significantly associated with the regulation of innate immunity and immune cells in AAA. Additionally, the role of aortic wall inflammation together with the immune infiltration in the abdominal aorta was reckoned as having high diagnostic and therapeutic potential of AAA (50, 51). As a result, we further conducted the immune infiltration analysis of AAA to explore the immune landscape of AAA as well as the correlation between key AAA-related m1A regulators and immune cells. Then, M0 macrophages, M1 macrophages, plasma cells and activated mast cells were identified as significant immune infiltrates cells in AAA. This result also reflected the diverse roles of macrophages polarization in AAA pathogenesis (24, 52, 53). However, due to the lack of relevant reports, the role of activated mast cells and plasma cells in AAA pathogenesis remains unclear. From this perspective, focusing on the relationship between macrophages and key AAA-related m1A regulators, we revealed a strong positive correlation between *YTHDF3* and M1/M2 macrophages and validated it in human AAA tissues. Therefore, in order to further explore the m1A regulation of macrophages polarization, *YTHDF3* and macrophages were both selected as the research subjects for our subsequent *in-vitro* experiments.

Through the analysis after M1 polarization of RAW264.7 cells and the knockdown of *ythdf3, YTHDF3* was verified to have a positive correlation with M1 macrophages and play an essential role in the promotion of macrophages M1 polarization. This finding provides a potential strategy of targeted therapy for AAA. To be specific, the specific knockdown of *ythdf3* in macrophages can effectively inhibit macrophage M1 polarization and facilitate M2 polarization. Therefore, the specific inhibitor of *YTHDF3* expression may serve as a modulator in the adaption of macrophage M2 polarization, which will decrease the secretion of matrix metalloproteinases (MMPs), facilitate reparative processes in aortic wall and attenuate the vascular inflammation by down-regulating the expression level of inflammatory cytokines like IL1β and TNF, up-regulating the secretion of anti-inflammatory cytokines and chemokines like IL10 and TGFβ (24, 54, 55). To sum up, *YTHDF3* has the potential to become a novel therapeutic target for AAA due to its modulation of macrophages polarization and the restoration of the M1 phenotype/M2 phenotype ratio at the site of AAA.

Lastly, we performed RIP-Seq analysis for *YTHDF3* and then used bioinformatics methods for PPI network analysis to dig the potential downstream target genes (DTGs) of *YTHDF3*. Thereafter, several DTGs of *YTHDF3* comprising *mTOR, CD44, ITGB1, ITGAVITGAV*, STAT1, and *STAT3*, etc. were identified to play potential roles in AAA formation and development. As for *CD44*, studies have proved that an enhanced expression of *CD44* exists in AAA, participating in the chronic vascular inflammation and the appearance of ectopic adipocytes in AAA wall (56, 57). Macrophages, as the major source of abundant *CD44*, can express various *CD44* variants, especially soluble *CD44*, to stimulate the expression and release of pro-inflammatory cytokine, IL1β, from ECs (56). It is also worth mentioning that novel chemo-photothermal synergistic therapy targeted on *CD44*-positive inflammatory macrophages is being developed for the treatment of atherosclerosis (58). In addition, *mTOR*, a signaling molecule also called the mechanistic target of rapamycin, has been identified as a key factor in the PI3K/Akt/*mTOR* or the AMPK/*mTOR* signaling pathway (59, 60). The overactivation of *mTOR* signaling accelerates AAA expansion *via* affecting the phenotype transition of VSMCs, macrophages infiltration, MMPs expression, and inflammatory cytokine production (61). Hence, *mTOR* is also reckoned as a potential target for AAA drug therapy including rapamycin, metformin (MET) and Gambogic acid (59, 60, 62). As for the integrin family (including *ITGB1, ITGAV, ITGA1*, etc.), Zheng et al. showed that the activation of integrin/*CD44* pathway will stimulate autophagy in VSMCs, facilitating the development of AAA (63). Scholars also found that the deficiency of specific integrin genes or the inhibition of integrin signaling pathways may ameliorate AAA progression by attenuating the infiltration of macrophages and decreasing the expression and activity of MMPs as well as reducing ECM degradation (64–67). Besides, the wide involvement of JAK-STAT pathway in AAA can modulate cytokine expression and immune cell activation, thus influencing the progression of AAA (68). Wu et al. and Xiao *et al*. independently pointed out that the blocking of *JAK2/STAT3* will result in the inhibition of experimental AAA growth through modulating aortic inflammation (69, 70). There are also reports about the role of *STAT1/STAT3* in the regulation of macrophages M1/M2 ratio (71–74), which imply the potential molecular mechanism underlying *YTHDF3*-modulated macrophage polarization, participating in the pathophysiological processes in AAA development.

Overall, our research is the first to observe that N1-Methyladenosine (m1A) regulators related genes are associated with the pathogenesis of abdominal aortic aneurysm through *YTHDF3* modulating macrophage polarization. Since the primary function of *YTHDF3* in macrophages is illustrated and the AAA-related target genes of *YTHDF3* are predicted, we believe the m1A regulation mechanism regarding *YTHDF3* will provide innovative potential targets for AAA therapy *via* adapting macrophage phenotypes. However, further research attempts are required to overcome the limitations of this study. For one thing, *in-vivo* experiments in animal models are required to confirm the regulatory effect of *YTHDF3* on macrophages polarization and AAA progression. For another, the detailed m1A epigenetic mechanism centered on the interaction between *YTHDF3* and other regulators, regulating the translation of target transcripts, needs to be explored through following research. Moreover, the m1A regulation involved in the interplays among macrophages, vascular endothelial cells (ECs), VSMCs and immune cells is also worth studying. Further research progress may shed some light on the role of m1A epigenetic regulation in AAA etiology as well as its promising future in the treatment of AAA.

## Conclusion

Our study for the first time systematically analyzed the expression pattern of m1A regulatory genes in human abdominal aortic aneurysms and identified the key AAA-related m1A regulators. The research results showed that *YTHDC1, YTHDF1* and *YTHDF3* may act as m1A “readers” to regulate their related genes *via* m1A modification, participating in the pathogenesis of human AAA. Moreover, the correlation between key AAA-related m1A regulators and immune cells were revealed by immune infiltration analysis in AAA. Thereafter, the significant correlation between *YTHDF3* and macrophages was verified in human AAA tissues. Now, we have found that *YTHDF3* may play an essential role in the pathogenesis of AAA by promoting macrophage pro-inflammatory M1 polarization. Our research provided novel insights into the etiology of AAA from the aspect of m1A epigenetic regulation of macrophage polarization. Our studies also paved the way for better understanding the molecular mechanism lying under *YTHDF3*-modulated macrophage polarization and exploring the genes targeted for m1A RNA methylation modifications that are able to impact AAA progression.

## Data Availability Statement

The datasets presented in this study can be found in online repositories. The names of the repository/repositories and accession number(s) can be found in the article/Supplementary Material.

## Ethics Statement

The studies involving human participants were reviewed and approved by the Ethics Committees of the First Hospital of China Medical University (Ethical Approval No: 2019-97-2). The patients/participants provided their written informed consent to participate in this study.

## Author Contributions

JZ and YH: conceptualization and funding acquisition. YW, FY, and SL: bioinformatics analysis. YW, DJ, HZ, and YY: experimental methodology. YW, HZ, and PG: data analysis. YW, DJ, and XZ: writing—original draft preparation. CB and CC: manuscript editing. DB, JZ, and YH: supervision. All authors have reviewed and approved the final version of the manuscript.

## Funding

This work was supported by the Fundamental Research Funds for the Central University (Grant No: DUT19RC(3)076), the National Natural Science Foundation of China (Grant No: 81600370), and the China Postdoctoral Science Foundation (Grant No: 2018M640270) for YH.

## Conflict of Interest

The authors declare that the research was conducted in the absence of any commercial or financial relationships that could be construed as a potential conflict of interest.

## Publisher's Note

All claims expressed in this article are solely those of the authors and do not necessarily represent those of their affiliated organizations, or those of the publisher, the editors and the reviewers. Any product that may be evaluated in this article, or claim that may be made by its manufacturer, is not guaranteed or endorsed by the publisher.

## Supplementary Material

Supplementary Table 1 The union set of co-expressed genes of DEMRGs.

Supplementary Table 2 The AAA-specific *YTHDF3*-binding mRNAs identified by RIP-Seq.

Supplementary Table 3 Node proteins in the PPI network.

Supplementary Figure 1 The GO/KEGG enrichment results of co-expressed genes of DEMRGs.

Supplementary Figure 2 The relative expression of *YTHDF3* in M0 macrophages and si-*ythdf3* macrophages, analyzed by RT-qPCR.
