# Supplementary figures and images for: N1-Methyladenosine (m1A) Regulation Associated With the Pathogenesis of Abdominal Aortic Aneurysm Through YTHDF3 Modulating Macrophage Polarization
Source: Front Cardiovasc Med. 2022 May 10;9:883155. doi: 10.3389/fcvm.2022.883155 (PMC9127271; doi:10.3389/fcvm.2022.883155)

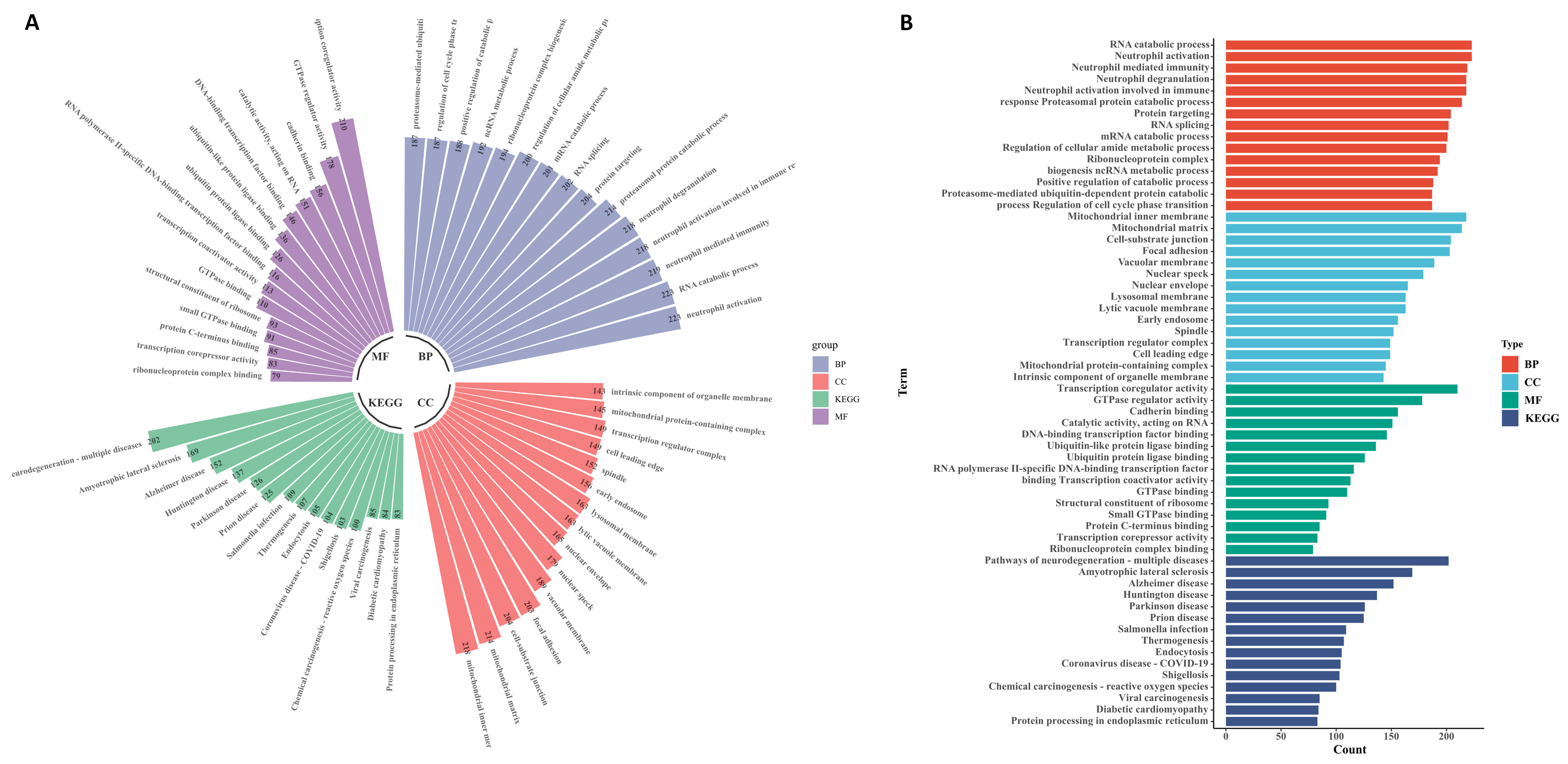

Supplement: Supplementary Figure 1 — The GO/KEGG enrichment results of co-expressed genes of DEMRGs. [file Image_1.png]

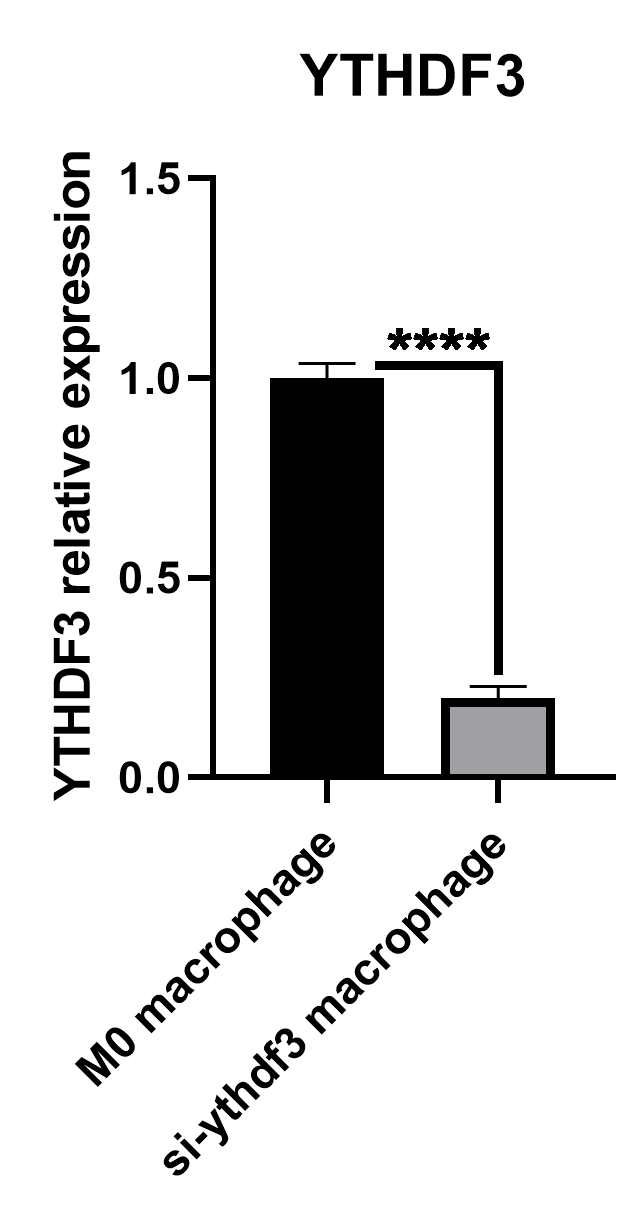

Supplement: Supplementary Figure 2 — The relative expression of YTHDF3 in M0 macrophages and si-ythdf3 macrophages, analyzed by RT-qPCR. [file Image_2.png]
